# Research Progress on Chemical Compositions, Pharmacological Activities, and Toxicities of Quinone Compounds in Traditional Chinese Medicines

**DOI:** 10.3390/toxics13070559

**Published:** 2025-06-30

**Authors:** Zhe Li, Rui Yao, Hong Guo, Wenguang Jing, Xiaohan Guo, Xiaoqiu Liu, Yingni Pan, Pei Cao, Lei Zhang, Jianbo Yang, Xianlong Cheng, Feng Wei

**Affiliations:** 1 School of Traditional Chinese Materia Medica, Shenyang Pharmaceutical University, Benxi 117004, China; lizhe0610@outlook.com (Z.L.);; 2 National Institutes for Food and Drug Control, Beijing 102629, China; 3 China National Center for Food Safety Risk Assessment, Beijing 100020, China

**Keywords:** quinones, chemical components, synthetic pathway, pharmacological activities, toxicity

## Abstract

With the continuous development of research on natural medicines, quinone compounds have become increasingly important in the research field of chemical constituents of natural treatments. However, there is a lack of in-depth and systematic collation of their types, distribution, pharmacological activities, and potential toxicities. This article comprehensively reviews the structural types, biogenetic pathways, extraction and separation methods, structural identification techniques, pharmacological activities, and toxicities of quinone compounds. It is found that the main difficulties in the research of quinone compounds lie in the cumbersome traditional separation and structural identification processes, as well as the insufficient in-depth studies on the mechanisms of their activities and toxicities. This review aims to provide a reference for research on quinone compounds in natural products and offer ideas and suggestions for subsequent in-depth exploration of the pharmacological activities of quinone compounds, prevention and control of their toxicities, and the realization of rational drug use.

## 1. Introduction

Quinone compounds are an important class of chemical constituents in natural medicines. They refer to natural organic compounds with an unsaturated cyclohexanedione structure within the molecule or are easily transformed into such structures [1]. According to the differences in their structures, quinone compounds are mainly classified into benzoquinones, naphthoquinones, phenanthraquinones, and anthraquinones [2], among which anthraquinones and their derivatives are the most numerous types. Quinone compounds are widely distributed in plants of families such as *Polygonaceae* Juss., *Rubiaceae* Juss., *Leguminosae* Lindl., *Rhamnaceae* Juss., and *Liliaceae* Juss., and are also present in the metabolites of some lower plants such as lichens and fungi. They possess various biological activities, including purgative, antibacterial, anti-tumor, diuretic, and hemostatic effects. In recent years, significant breakthroughs have been achieved in the research and development of new drugs derived from natural quinone compounds across multiple fields. For example, sodium tanshinone IIA sulfonate, a drug for treating coronary heart disease [3], and buparvaquone, an antimalarial drug [4]. In recent years, remarkable progress has been made in the research on the pharmacological activities of quinone chemical constituents in traditional Chinese medicines. However, quinone compounds have many adverse reactions, such as hepatotoxicity, nephrotoxicity, and carcinogenicity, which require widespread attention. Traditional Chinese medicines containing anthraquinone components may cause adverse reactions, such as melanosis coli, drug-induced liver injury, and drug-induced kidney injury in clinical practice [5]. Zhou Xujun’s analysis of 130 patients with melanosis coli showed that among 108 patients with constipation, 97 had a history of taking anthraquinone laxatives. Among them, 73 patients were grade III, and the medication duration was 1–4 years [6]. Wang Xiong [7] conducted a retrospective analysis of 12 inpatients with drug-induced liver injury caused by taking *Pleuropterus multiflorus* (Thunb.) Nakai and its related preparations were admitted to the Department of Hepatology of the First Affiliated Hospital of Hunan University of Chinese Medicine from January 2017 to March 2024. The severity classification was as follows: 8 cases; grade 1, 3 cases; and grade 3, and 1 case was at grade 4. All patients with grade 3 and above liver injury received traditional Chinese medicine prescriptions, and liver injury in those who took proprietary Chinese medicines was mostly mild. After discontinuing the related preparations and receiving symptomatic supportive treatments, such as liver protection and transaminase level reduction, all patients improved and were discharged from the hospital. The occurrence of drug-induced kidney injury may be related to *Aloe vera* (Haw.) Berg, *Senna alexandrina* Mill., *Astragalus membranaceus* (Fisch.) Bunge, *Reynoutria japonica* Houtt., *Senna obtusifolia* (L.) H. S. Irwin and Barneby [8]. Zhao Fengbo [9] analyzed 172 patients with renal parenchymal acute kidney injury (AKI). The results showed that 39 cases were caused by the consumption of Chinese herbal medicines. Among the causative herbal medicines, *Aloe vera* (Haw.) Berg containing anthraquinone components was included.

**Figure 1:**
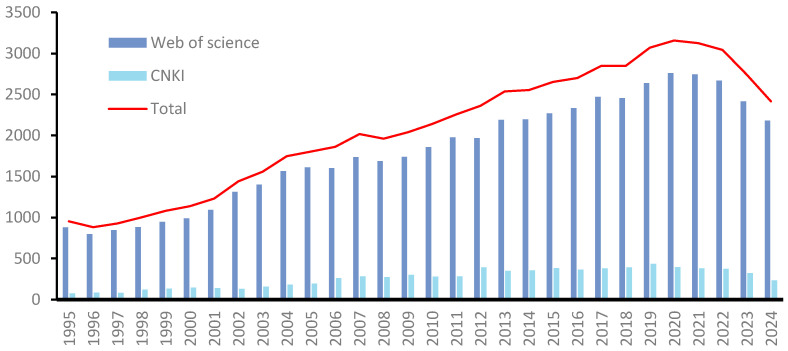
The number of references based on quinones.

The dynamic changes in the number of literature, to a certain extent, reflect the academic community’s attention and research progress on quinone compounds. This article conducted searches in the China National Knowledge Infrastructure (CNKI) and Web of Science databases. In CNKI, the advanced search method was adopted, with “quinones (exact)” as the search term; in the Web of Science, the search condition is set as: Topic = “quinone”. The search period was set from 1995 to 2024. After the search, 62,241 literature were obtained, among which 7927 were included in CNKI and 54,314 were included in the Web of Science. The number of references on quinone compounds has generally shown an upward trend. An increasing number of new quinone compounds have been extracted, separated, and identified, and their pharmacological activities and synthesis pathways have been further elucidated. This review elaborates on the chemical constituents, synthesis pathways, pharmacological activities, and toxicities of quinone compounds in traditional Chinese medicine, with the aim of providing scientific references for subsequent research on the pharmacological activities of quinone compounds, toxicity prevention and control, and safety evaluation standards.Figure 1 introduces the number of references based on quinone compounds.

## 2. Progress in Chemical Composition Research

### 2.1. Structure Type and Distribution

#### 2.1.1. Benzoquinones

**Figure 2:**
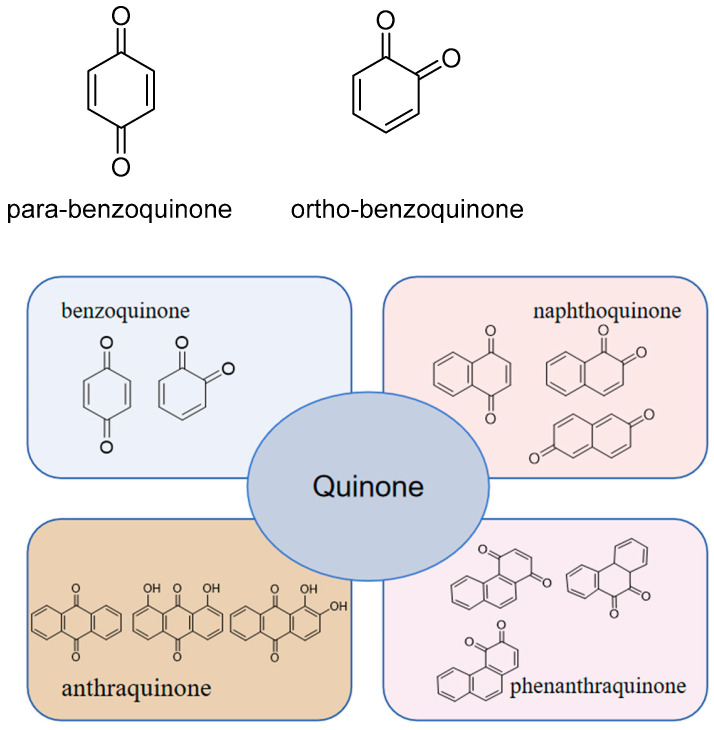
Classification of the skeletal structures of quinone compounds.

Benzoquinones are structurally divided into two major groups: ortho-benzoquinone and para-benzoquinone, and compounds with pro-benzoquinone structures are unstable; therefore, most naturally occurring benzoquinone compounds are para-benzoquinone derivatives [10]. The substituents of benzoquinone are more varied and are usually classified into small and large groups. Common small groups include hydroxyl, methoxy, carboxyl, and smaller hydrocarbon groups containing less than three carbons, while large groups include saturated or unsaturated chain hydrocarbons containing more than three carbon atoms, benzene rings, and more complex carbon-containing substituents. Figure 2 introduces the classification of the skeletal structures of quinone compounds.

Benzoquinones can be categorized into small-molecule benzoquinones, advanced straight-chain hydrocarbon benzoquinones, isopentenyl benzoquinones, furanobenzoquinones, flavonoid benzoquinones, terpene benzoquinones, and benzoquinones based on the nature of the substituent groups [11]. Small-molecule benzoquinones are common small-molecule substituents, such as hydroxyl, methoxy, and alkyl groups, which are attached to the parent nucleus of the benzoquinone. A total of 14 types of small-molecule benzoquinones have been identified, and examples of small-molecule benzoquinones include 2-methyl-p-quinone, 2, 6-dimethoxy-1, 4-benzoquinone, and others. Advanced straight-chain hydrocarbon benzoquinones have at least one advanced straight-chain aliphatic hydrocarbon attached to the parent nucleus of the benzoquinone, and nine types have been found, such as primin and arnebifuranone. Isopentenyl benzoquinones have a variable number of isopentenyl groups attached to the parent nucleus of the benzoquinone, of which 12 have been found, such as omphalone, 3-bydroxy-2-methyl-5-(3-methyl-2-butenyl)benzo-1,4-quinone. Furobenzoquinones are compounds formed by the fusion of a benzoquinone with a furan ring, of which there are three. An example of a furan-based benzoquinone is cyperaquinone. Flavonoid benzoquinones are structurally characterized by a skeleton similar to that of flavonoids, with the difference that the B ring of this class of compounds is not a benzene ring, but a benzoquinone and its derivatives, of which there are four, such as cyclofissoquinone and bodimoquinone. Terpene quinones are compounds with a terpene skeleton but with a benzoquinone structure in the molecule. There are four kinds, such as 3-acetoxymo-quinone. Biphenylquinone is a dimer consisting of two identical or different benzoquinones linked by a carbon-carbon bond, there are seven kinds. Examples of biphenylquinones include methylvilangin and lanciaquinone.

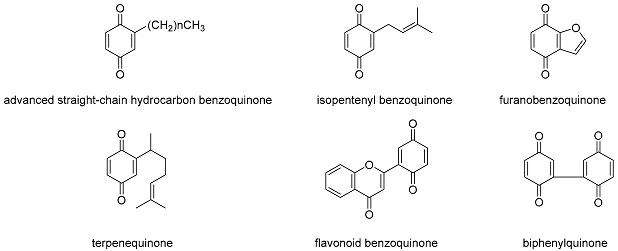



**Figure 3:**
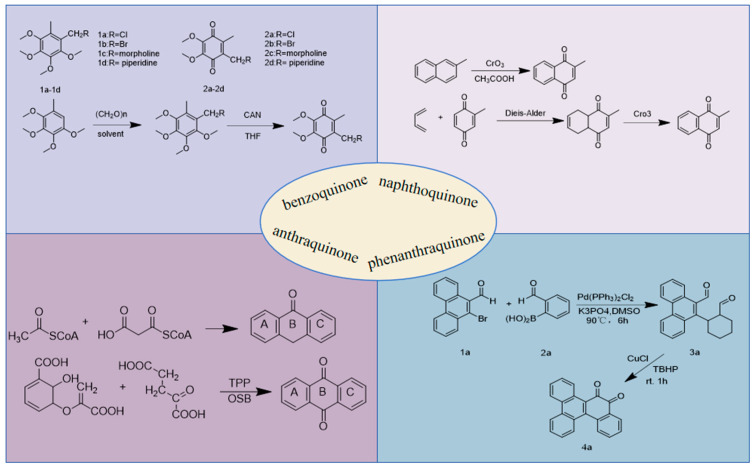
Synthetic pathways of quinone compounds.

1,4 Benzoquinone was synthesized using a two-step process. In the first step, compound 1 was reacted with paraformaldehyde in different solvents (37% hydrochloric acid, 47% hydrogen bromide, morpholine, and piperidine) for 2 h at 35 °C to give compounds **2a**–**2d** in high yields. The second step involved the oxidation of compounds **1a**–**1d** with cerium ammonium nitrate (CAN) at room temperature to obtain the desired compounds **2a**–**2d** in good yields. This method is short, high-yield, and easy to post-process [12]. Figure 3 introduces the synthetic pathways of quinone compounds.

Benzoquinones are found in *Leguminosae* Lindl., *Asteraceae* L., *Comfreyaceae* L., Araceae Juss., and some fungi. Among them, four isopentenyl-substituted benzoquinones were isolated from *Nephthea chabrolii* Audouin, one small-molecule benzoquinone, one high-level straight-chain hydrocarbon benzoquinone, and two isopentenyl-substituted benzoquinones from *Arnebia euchroma* (Royle) I.M. Johnst., and four small-molecule benzoquinones from *Antrodia cinnamomea* T. T. Chang & W. N. Chou. Three flavonoid benzoquinones were isolated from *Dalbergia odorifera* T. Chen. Two biphenoquinones and one advanced straight-chain hydrocarbon benzoquinone were isolated from *Myrsine africana* L. var. *acuminata* C. Y. Wu et C. Chen (synonym). Two isopentenyl-substituted benzoquinones were isolated from *Atractylodes koreana* (Nakai) Kita. Two terpene benzoquinones were isolated from *Helianthus annuus* L. Two advanced straight-chain hydrocarbon benzoquinones were isolated from *Embelia ribes* Burm. f. Table 1 presents the names and molecular formulas of benzoquinone compounds.

**Table 1:** Names and molecular formulas of the benzoquinone compounds.

No.	Name	Resource	Molecular	Classification	Ref.
1	2-methyl-p-quinone	*Blaps rynchopetera* Fairmaire	C_7_H_6_O_2_	small molecule benzoquinone	[13]
2	2,5-dimethyl-3-methoxy-p-benzoquinone	*Fluridobulus penneri*	C_9_H_10_O_3_	small molecule benzoquinone	[14]
3	2, 6-dimethoxy-1, 4-benzoquinone	*Atractylodes macrocephala* Koidz	C_8_H_8_O_4_	small molecule benzoquinone	[15]
4	aurantiogliocladin	*Arnebia euchroma* (Royle) I.M. Johnst.	C_10_H_12_O_4_	small molecule benzoquinone	[16]
5	2-hydroxy-3-methoxy-5-methyl-p-benzoquinone	*Antrodia cinnamomea* T. T. Chang & W. N. Chou	C_8_H_8_O_4_	small molecule benzoquinone	[17]
6	2-methoxy-6-methyl-p-benzoquinone	*Antrodia cinnamomea* T. T. Chang & W. N. Chou	C_8_H_8_O_3_	small molecule benzoquinone	[17]
7	2,3-dimethoxy-5-methyl-p-benzoquinone	*Antrodia cinnamomea* T. T. Chang & W. N. Chou	C_9_H_10_O_4_	small molecule benzoquinone	[17]
8	2-hydroxy-5-methoxy-3-methyl-p-benzoquinone	*Antrodia cinnamomea* T. T. Chang & W. N. Chou	C_8_H_8_O_4_	small molecule benzoquinone	[17]
9	anserinone A	*Podospora anserina* (Rabenh.) Niessl	C_11_H_12_O_4_	small molecule benzoquinone	[18]
10	anserinone B	*Podospora anserina* (Rabenh.) Niessl	C_11_H_14_O_4_	small molecule benzoquinone	[18]
11	2-hydroxy-3-methyl-5-methoxy-p-benzoquinone	*Pterospermum heterophyllum* Hance	C_8_H_8_O_4_	small molecule benzoquinone	[14]
12	2.3-dimethyl-5, 6-dimethoxy-p-benzoquinone	*Gliocladium penicilloides* Corda	C_10_H_12_O_4_	small molecule benzoquinone	[14]
13	2, 5-dimethoxy-3, 6-dimethyl-p-benzoquinone	*Neonectria fuckeliana* (C. Booth) Castl. & Rossman	C_10_H_12_O_4_	small molecule benzoquinone	[14]
14	thymoquinone	*Nigella sativa* L.	C_10_H_12_O_2_	small molecule benzoquinone	[19]
15	primin	*Miconia lepidota* DC.	C_12_H_16_O_3_	advanced straight-chain hydrocarbon benzoquinone	[20]
16	embelin	*Embelia ribes* Burm. f	C_17_H_26_O_4_	advanced straight-chain hydrocarbon benzoquinone	[21]
17	2,5-dihydroxy-3-tridecyl-1, 4-benzoquinone	*Embelia ribes* Burm. f.	C_19_H_30_O_4_	advanced straight-chain hydrocarbon benzoquinone	[21]
18	myrsinone	*Myrsine africana* L. var. *acuminata* C. Y. Wu et C. Chen (synonym)	C_17_H_26_O_4_	advanced straight-chain hydrocarbon benzoquinone	[14]
19	idebenone	-	C_19_H_30_O_5_	advanced straight-chain hydrocarbon benzoquinone	[22]
20	2-methoxy-6-nonadecyl-1,4-benzoquinone	*Miconia lepidota* DC.	C_26_H_44_O_3_	advanced straight-chain hydrocarbon benzoquinone	[23]
21	(-)-a-tocospirone	*Gynura japonica* (Thunb.) Juel	C_29_H_50_O_4_	advanced straight-chain hydrocarbon benzoquinone	[24]
22	maesaquinone	*Maesa japonica* (Thunb.) Moritzi	C_26_H_42_O_4_	advanced straight-chain hydrocarbon benzoquinone	[25]
23	paphionone	*Paphiopedilum exul* (Ridl.) Rolfe	C_20_H_30_O_5_	advanced straight-chain hydrocarbon benzoquinone	[26]
24	isopentenyl p-benzoquinone	*Phagnalon purpurescens* Sch. Bip.	C_11_H_12_O_2_	isopentenyl benzoquinone	[14]
25	3,5,6-trimethoxy-2-isopentene-p-benzoquinone	*Dendrobium nobile* Lindl.	C_14_H_18_O_5_	isopentenyl benzoquinone	[14]
26	omphalone	*Lentinellus micheneri* (Berk. & M. A. Curtis) Pegler	C_11_H_8_O_3_	isopentenyl benzoquinone	[27]
27	2(E) -2-geranyl-6-methyl p-benzoquinone	*Atractylodes koreana* (Nakai) Kita.	C_17_H_22_O_2_	isopentenyl benzoquinone	[14]
28	2-(Z) -2-geranyl-6-methyl p-benzoquinone	*Atractylodes koreana* (Nakai) Kita.	C_17_H_22_O_2_	isopentenyl benzoquinone	[14]
29	amebifuranone	*Arnebia euchroma* (Royle) I.M. Johnst	C_18_H_20_O_5_	isopentenyl benzoquinone	[14]
30	arnebinone	*Arnebia euchroma* (Royle) I.M. Johnst	C_18_H_22_O_4_	isopentenyl benzoquinone	[14]
31	chabrolobenzoquinone E	*Nephthea chabrolii* Audouin	C_27_H_38_O_3_	isopentenyl benzoquinone	[28]
32	chabrolobenzoquinone F	*Nephthea chabrolii* Audouin	C_29_H_40_O_4_	isopentenyl benzoquinone	[28]
33	chabrolobenzoquinone G	*Nephthea chabrolii* Audouin	C_27_H_38_O_3_	isopentenyl benzoquinone	[28]
34	chabrolobenzoquinone H	*Nephthea chabrolii* Audouin	C_29_H_42_O_5_	isopentenyl benzoquinone	[28]
35	atrovirinone	*Garcinia atroviridis* Griffith ex T. Anderson	C_25_H_28_O_8_	isopentenyl benzoquinone	[29]
36	cyperaquinone	*Cyperus nipponicus* Franch. & Sav.	C_14_H_10_O_4_	furanobenzoquinone	[30]
37	albidin	*Penicillium albidum* Sopp	C_10_H_8_O_4_	furanobenzoquinone	[14]
38	graphisquinone	*Graphis scripta* (L.) Ach.	C_11_H_10_O_5_	furanobenzoquinone	[14]
39	chrysoquinane	*Euphorbia esula* L.	C_19_H_16_O_9_	flavonoid benzoquinone	[14]
40	claussequinone	*Dalbergia odorifera* T.Chen	C_16_H_16_O_5_	flavonoid benzoquinone	[14]
41	bowdichione	*Dalbergia odorifera* T.Chen	C_16_H_10_O_6_	flavonoid benzoquinone	[14]
42	donoherbivol-cyclocledoquinone	*Dalbergia odorifera* T.Chen	C_32_H_28_O_9_	flavonoid benzoquinone	[14]
43	3-Acetoxymo-quinone	*Cordia oncocalyx* (Allemão) Baill.	C_12_H_14_O_4_	terpenebenzoquinone	[31]
44	glanduline A	*Helianthus annuus* L.	C_15_H_20_O_2_	terpenebenzoquinone	[14]
45	glanduline B	*Helianthus annuus* L.	C_15_H_18_O_2_	terpenebenzoquinone	[14]
46	methylvilangin	*Myrsine africana* L. var. *acuminata* C. Y. Wu et C. Chen (synonym)	C_36_H_54_O_8_	biphenylquinone	[25]
47	methylanhydrovilangin	*Myrsine africana* L. var. *acuminata* C. Y. Wu et C. Chen (synonym)	C_16_H_52_O_7_	biphenylquinone	[25]
48	lanciaquinone	*Ardisia japonica* (Thunb.) Bl.	C_27_H_36_O_7_	biphenylquinone	[32]
49	neonambiquinone A	*Neonothopanus nambi* (Speg.) R. H. Petersen & Krisai	C_19_H_14_O_6_	biphenylquinone	[33]
50	volucrisporin	*Volucrispora aurantiaca* Haskins	C_18_H_12_O_4_	biphenylquinone	[34]
51	oosporein	*Beauveria bassiana* (Bals.-Criv.) Vuill.	C_14_H_18_O_8_	biphenylquinone	[35]
52	biembelin	*Rapanea melanophloeos* (L.) Meisn.	C_34_H_50_O_8_	biphenylquinone	[14]
53	embenones A	*Knema globularia* (Lam.) Warb.	C_15_H_18_O_4_	other	[35]
54	embenones B	*Knema globularia* (Lam.) Warb.	C_15_H_20_O_4_	other	[35]
55	triaziquone	*Artemisia sieberi*. J	C_12_H_13_N_3_O_2_	other	[36]
56	aziridyl benzoquinone	-	C_16_H_22_N_2_O_6_	other	[37]
57	erectquione B	*Hypericum erectum* Sol. ex R.Br.	C_29_H_40_O_6_	other	[38]
58	erectquione C	*Hypericum erectum* Sol. ex R.Br.	C_25_H_34_O_6_	other	[38]
59	Atromentin	*Ascocoryne sarcoides*	C_18_H_12_O_6_	other	[39]
60	Erectquione A	*Hypericum erectum* Sol. ex R.Br.	C_21_H_28_O_4_	ortho-benzoquinone	[38]

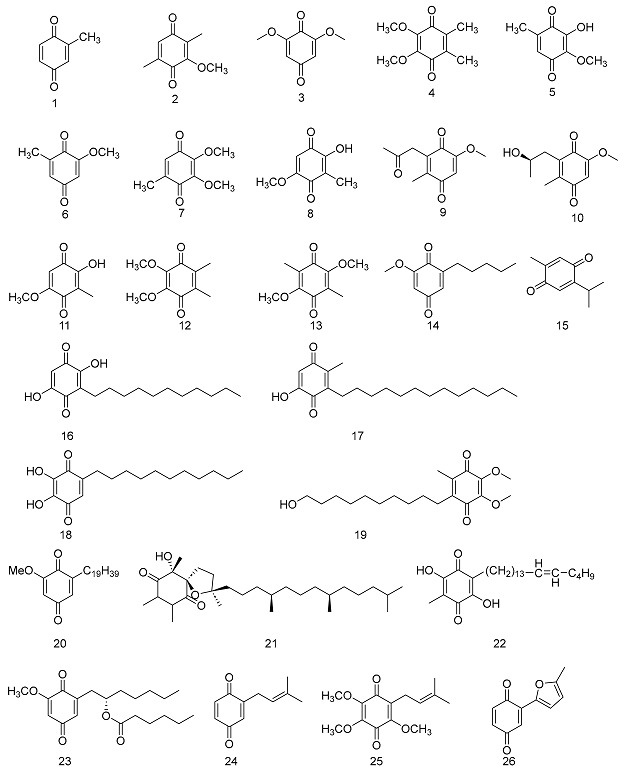

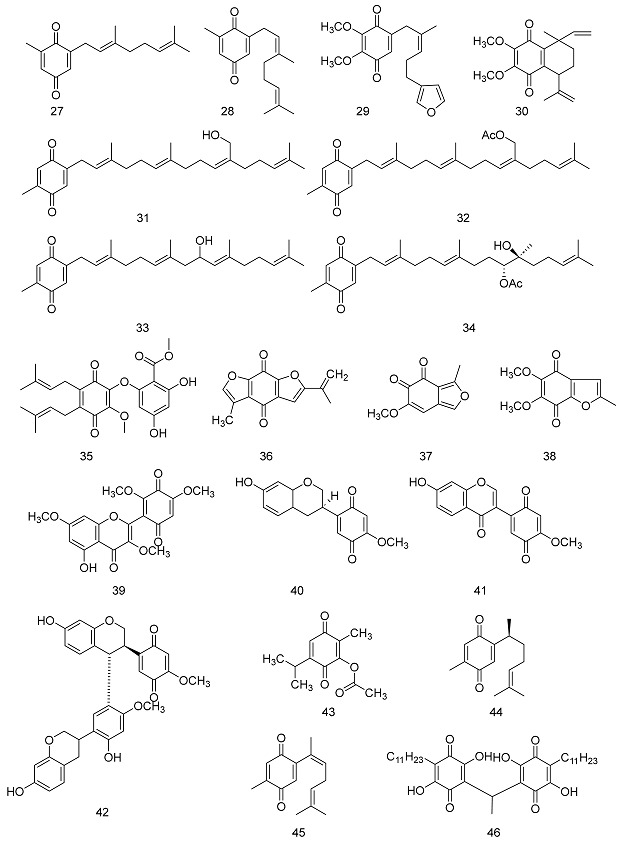

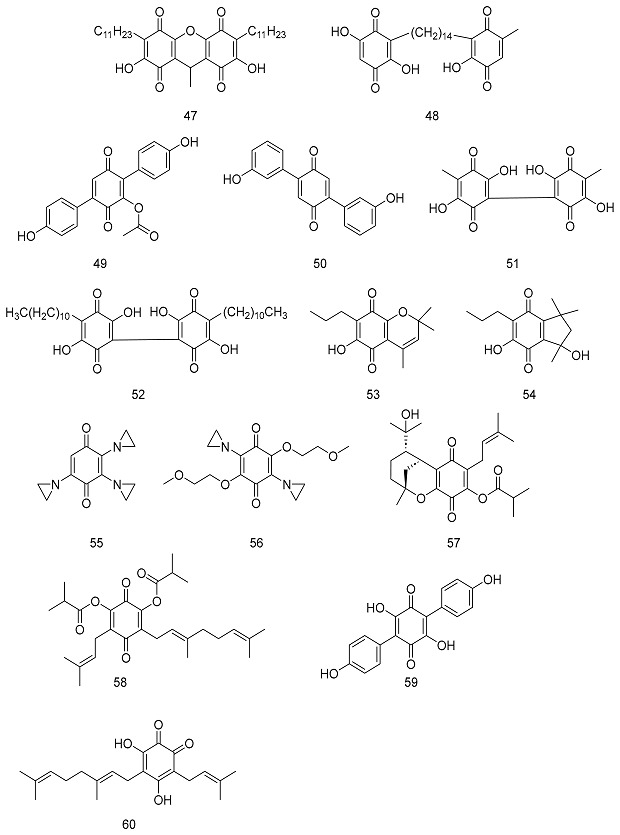







#### 2.1.2. Naphthoquinones

Naphthoquinones can be structurally divided into three types: α(1,4) naphthoquinone, β(1,2) naphthoquinone, and amphi(2,6) naphthoquinone, of which most naturally occurring naphthoquinones are α-naphthoquinone derivatives [40]. They are mostly orange or orange-red crystals, and a few are purple.

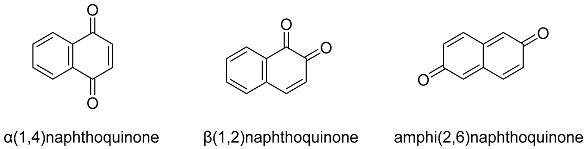



Common naphthoquinone substituents include hydroxyl, methoxy, aliphatic, and aromatic hydrocarbons. Naphthoquinones can be categorized based on the type of substituents as small-molecule-substituted naphthoquinones, benzoisochromanquinones, furanonaphthoquinones, isopentenyl naphthoquinones, etc. [11]. Small-molecule naphthoquinones are common small-molecule substituents, such as hydroxyl, methoxy, and alkyl groups, attached to the parent nucleus of naphthoquinone. Currently, 24 small-molecule-substituted naphthoquinones have been identified, including juglone and plumbagin; 20 benzoisochroman quinones, including davidianone A and mansonin A; 28 furano-naphthoquinones, including arthoniafurone B and cribrarione A; and 23 isopentenyl naphthoquinones, including lapachol and crassiflorone.

There are two mainstream methods for synthesizing 2-methyl-1,4-naphthoquinone. The first method uses 2-methylnaphthalene as the raw material and glacial acetic acid as the solvent, and 2-methyl-1,4-naphthoquinone is obtained via one-step oxidation with chromium trioxide. The main advantage of this method is that 2-methylnaphthalene is inexpensive, and the route is only one step. 2-Methylnaphthalene hydroquinone is obtained by Diels-Alder cycloaddition of butadiene and methylbenzoquinone, followed by oxidation with chromic anhydride to obtain 2-methyl-1,4-naphthoquinone [41].

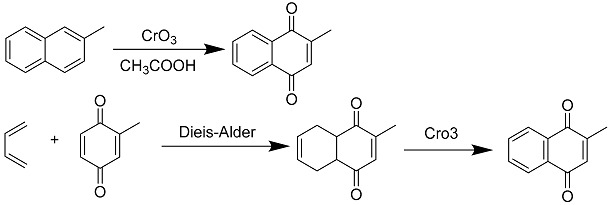



Naphthoquinones are mainly distributed in plants of the families *Ulmaceae* Mirb., *Persicaceae* Raf., and *Albiziaceae* Raf., in addition to some microorganisms and marine organisms. Among them, 20 naphthoquinones were isolated from *Rhinacanthus nasutus* (L.) Kurz, containing six benzoisochromanquinones and eight isoprenoid naphthoquinones; 7 naphthoquinones were isolated from *Cordia curassavica* (Jacq.) Roem. & Schult; Five naphthoquinones, containing one small-molecule naphthoquinone, and three furanoquinones were isolated from *Plumbago zeylanica* L.; four naphthoquinones were isolated from *Chirita eburnea* Hance; four benzoisochromanquinones were isolated from *Ulmus pumila* L.; three small-molecule naphthoquinones were isolated from *Diospyros maritima* Blume; three small-molecule naphthoquinones and three benzisochromanquinones were isolated from *Ulmus davidiana* Planch. Table 2 presents the names and molecular formulas of naphthoquinone compounds.

**Table 2:** Names and molecular formulas of naphthoquinone compounds.

No.	Name	Resource	Formula	Classification	Ref.
61	3-bromoplumbagin	*Diospyros maritima* Blume	C_11_H_7_BrO_3_	small molecule naphthoquinones	[42]
62	3-(2-hydroxyethyl)plumbagin	*Diospyros maritima* Blume	C_13_H_12_O_4_	small molecule naphthoquinones	[42]
63	6-(1-ethoxyethyl)plumbagin	*Diospyros maritima* Blume	C_15_H_16_O_4_	small molecule naphthoquinones	[43]
64	juglone	*Juglans regia* L.	C_10_H_6_O_3_	small molecule naphthoquinones	[14]
65	2-methyl-1, 4-naphthoquinone	*Juglans regia* L.	C_11_H_8_O_2_	small molecule naphthoquinones	[14]
66	lawsone	*Lythrum salicaria* L.	C_10_H_6_O_4_	small molecule naphthoquinones	[14]
67	2-amino-1.4-naphthoquinone	*Laurus nobilis* L.	C_10_H_7_NO_3_	small molecule naphthoquinones	[14]
68	plumbagin	*Plumbago zeylanica* L.	C_11_H_8_O_3_	small molecule naphthoquinones	[14]
69	isoplumbagin	*Impatiens balsamina* L.	C_11_H_8_O_3_	small molecule naphthoquinones	[14]
70	chimaphilin	*Pyrola soldanellifolia* Andres	C_12_H_10_O_3_	small molecule naphthoquinones	[14]
71	7-methyl juglone	*Diospyros usambarensis* Engl.	C_11_H_8_O_3_	small molecule naphthoquinones	[14]
72	2-methoxy-6-acetyl-7-methyljuglone	*Pleuropterus multiflorus* (Thunb.) Nakai	C_13_H_12_O_5_	small molecule naphthoquinones	[44]
73	2-methoxystypandrone	*Rumex japonicus* Houtt	C_14_H_12_O_5_	small molecule naphthoquinones	[45]
74	2-butanoyl-3,6,8-trihydroxy-1,4-naphthoquinone 6-*O*-sulfate	*Oxycomanthus japonicus* J. F. W. Mller	C_14_H_11_NaO_9_S	small molecule naphthoquinones	[46]
75	2-butanoyl-3,6,8-trihydroxy-1,4-naphthoquinone	*Oxycomanthus japonicus* J. F. W. Mller	C_14_H_12_O_6_	small molecule naphthoquinones	[46]
76	cribrarione B	*Cribraria cancellata* (Batsch) Nann.-Bremek.	C_12_H_10_O_6_	small molecule naphthoquinones	[47]
77	fusarnaphthoquinoe A	*Fusarium* spp.	C_15_H_18_O_7_	small molecule naphthoquinones	[48]
78	7-carbomethoxy-2,8-dimethoxy-5-hydroxy-l,4-naphthoquinone	*Penicillium raistrickii* Stolk & Scott	C_14_H_13_O_7_	small molecule naphthoquinones	[49]
79	2,7-dimethoxy-5-hydroxy-1,4-naphthoquinone	*Penicillium raistrickii* Stolk & Scott	C_12_H_10_O_5_	small molecule naphthoquinones	[49]
80	8-formyl-7-hydroxy-5-isopropyl-2-methoxy-3-methyl-1,4-naphthoquinone	*Ceiba pentandra* (L.) Gaertn.	C_16_H_16_O_5_	small molecule naphthoquinones	[50]
81	2,7-dihydroxy-8-formyl-5-isopropyl-3-methyl-1.4-naphthoquinone	*Ceiba pentandra* (L.) Gaertn.	C_15_H_14_O_5_	small molecule naphthoquinones	[50]
82	7-hydroxy-5-isopropyl-2-methoxy-3-methylnaphthoquinone	*Bombax malabaricum* DC.	C_15_H_16_O_4_	small molecule naphthoquinones	[51]
83	lanigerone	*Salvia lanigera* Poir. (Lamiaceae)	C_14_H_14_O_3_	small molecule naphthoquinones	[52]
84	salvigerone	*Salvia lanigera* Poir. (Lamiaceae)	C_21_H_26_O_4_	small molecule naphthoquinones	[52]
85	droserone	*Plumbago capensis* Thunb	C_11_H_8_O_4_	small molecule naphthoquinones	[53]
86	davidianone A	*Ulmus davidiana* Planch.	C_15_H_12_O_4_	benzoisochromanquinone	[54]
87	davidianone B	*Ulmus davidiana* Planch.	C_16_H_12_O_5_	benzoisochromanquinone	[54]
88	davidianone C	*Ulmus davidiana* Planch.	C_17_H_16_O_5_	benzoisochromanquinone	[54]
89	mansonone E	*Ulmus pumila* L.	C_15_H_14_O_3_	benzoisochromanquinone	[55]
90	mansonone F	*Ulmus pumila* L.	C_15_H_12_O_3_	benzoisochromanquinone	[55]
91	mansonone H	*Ulmus pumila* L.	C_15_H_14_O_4_	benzoisochromanquinone	[56]
92	mansonone I	*Ulmus pumila* L.	C_15_H_14_O_4_	benzoisochromanquinone	[57]
93	rhinacanthone	*Rhinacanthus nasutus* (L.) Kurz	C_15_H_14_O_3_	benzoisochromanquinone	[58]
94	rhinacanthin A	*Rhinacanthus nasutus* (L.) Kurz	C_15_H_14_O_4_	benzoisochromanquinone	[59]
95	rhinacanthin O	*Rhinacanthus nasutus* (L.) Kurz	C_24_H_26_O_5_	benzoisochromanquinone	[58]
96	rhinacanthin P	*Rhinacanthus nasutus* (L.) Kurz	C_24_H_26_O_5_	benzoisochromanquinone	[58]
97	rhinacanthin S	*Rhinacanthus nasutus* (L.) Kurz	C_24_H_24_O_5_	benzoisochromanquinone	[58]
98	rhinacanthin T	*Rhinacanthus nasutus* (L.) Kurz	C_24_H_26_O_5_	benzoisochromanquinone	[60]
99	mansonin A	*Mansonia altissima* A. Chev.	C_17_H_18_O_5_	benzoisochromanquinone	[60]
100	mansonin B	*Mansonia altissima* A. Chev.	C_17_H_18_O_6_	benzoisochromanquinone	[60]
101	5-methoxy-3,4-dehydroxanthomegnin	*Paepalanthus latipes* Silveira	C_16_H_12_O_7_	benzoisochromanquinone	[61]
102	pyranokunthone A	*Stereospermum kunthianum* Cham.	C_20_H_20_O_4_	benzoisochromanquinone	[62]
103	4-*O*-methyl erythrostominone	*Cordyceps unilateralis* (Tul.) Sacc. var. *clavata* (Y. Kobayasi)	C_18_H_18_O_8_	benzoisochromanquinone	[63]
104	halawanone A	*Streptomyces* Schröter	C_23_H_22_O_9_	benzoisochromanquinone	[64]
105	pyranokunthone B	*Stereospermum kunthianum* Cham.	C_20_H_20_O_4_	benzoisochromanquinone	[62]
106	(3a,3′a,4β,β)-3,3′-dimethoxy-cis-[4,4′-bis(3,4,5,10-tetra-hydro-1*H*-naphtho(2,3-clpyran)]-5.5.10,10-tetraone	*Pentas longiflora* Oliv.	C_28_H_22_O_8_	benzoisochromanquinone	[65]
107	arthoniafurone B	*Arthonia cinnabarina* Ach.	C_14_H_10_O_5_	furanonaphthoquinone	[66]
108	fusarnaphthoquinone B	*Fusarium* Link	C_15_H_16_O_5_	furanonaphthoquinone	[48]
109	arthoniafurone A	*Arthonia cinnabarina* (DC.) Wallr.	C_14_H_8_O_5_	furanonaphthoquinone	[66]
110	cribrarione A	*Cribraria purpurea* Schwein.	C_13_H_10_O_7_	furanonaphthoquinone	[67]
111	8-hydroxy-1-methylnaphtho[2,3-*c*]furan-4,9-dione	*Bulbine capitata* Poelln.	C_13_H_8_O_4_	furanonaphthoquinone	[68]
112	5,8-dihydroxy-1-methylnaphtho[2,3-*c*]furan-4,9-dione	*Aloe ferox* Mill.	C_13_H_8_O_5_	furanonaphthoquinone	[69]
113	5,8-dihydroxy-1-hydroxymethylnaphtho[2,3-*c*]furan-4,9-dione	*Aloe ferox* Mill.	C_13_H_8_O_6_	furanonaphthoquinone	[69]
114	avicequinone A	*Avicennia alba* Blume	C_15_H_14_O_5_	furanonaphthoquinone	[70]
115	avicequinone B	*Avicennia alba* Blume	C_12_H_6_O_3_	furanonaphthoquinone	[70]
116	avicequinone C	*Avicennia alba* Blume	C_15_H_12_O_4_	furanonaphthoquinone	[70]
117	avicequinone D	*Avicennia alba* Blume	C_15_H_12_O_5_	furanonaphthoquinone	[70]
118	avicequinone E	*Mendoncia cowanii* (S. Moore) Benoist	C_15_H_14_O_5_	furanonaphthoquinone	[71]
119	2-(1′-methylethenyl)naphtho[2,3-*b*]furan-4,9-dione	*Newbouldia laevis* (P. Beauv.) Seem. ex Bureau	C_15_H_10_O_3_	furanonaphthoquinone	[72]
120	2-isopropenyl-9-methaxy-1,8-dioxa-dicyclopenta[*b*,*g*]naphthal-ene-4,10-dione	*Plumbago zeylanica* L.	C_18_H_12_O_5_	furanonaphthoquinone	[73]
121	9-hydroxy-2-isopropenyl-1,8-dioxa-dicyclopenta[*b*,*g*]naphthal-ene-4,10-dione	*Plumbago zeylanica* L.	C_17_H_10_O_5_	furanonaphthoquinone	[74]
122	2-(1-hydroxy-l-methyl-ethyl)-9-methoxy-1,8-dioxa-dicyclo-penta[b,g]naphthalene-4,10-dione	*Plumbago zeylanica* L.	C_18_H_14_O_6_	furanonaphthoquinone	[73]
123	(*R*)-7-hydroxy-*a*-dunnione	*Chirita eburnea* Hance	C_15_H_14_O_4_	furanonaphthoquinone	[74]
124	(*R*)-8-hydroxy-*a*-dunnione	*Chirita eburnea* Hance	C_15_H_14_O_4_	furanonaphthoquinone	[74]
125	(*R*)-*a*-7,8-dihydroxy-*a*-dunnione	*Chirita eburnea* Hance	C_15_H_14_O_5_	furanonaphthoquinone	[74]
126	(*R*)-7-methoxy-6,8-dihydroxy-*a*-dunnione	*Chirita eburnea* Hance	C_16_H_16_O_6_	furanonaphthoquinone	[74]
127	7,8-dimethoxydunnione	*Sinningia leucotricha* (Hoehne) H. E. Moore	C_17_H_18_O_5_	furanonaphthoquinone	[75]
128	dehydro-*a*-isodunnione	*Tectona grandis* L. f.	C_15_H_12_O_3_	furanonaphthoquinone	[76]
129	5-hydroxy-7-methoxydehydroiso-*a*-lapachone	*Newbouldia laevis* (P. Beauv.) Seemann ex Bureau	C_16_H_14_O_5_	furanonaphthoquinone	[77]
130	glycoquinone	*Glycosmis pentaphylla* (Retz.) Corrêa	C_20_H_24_O_4_	furanonaphthoquinone	[78]
131	(2R)-6,8-dihydroxy-a-dunnione	*Lysionotus pauciflorus* Maxim.	C_15_H_14_O_5_	furanonaphthoquinone	[79]
132	balsaminone D	*Impatiens balsamina* L.	C_20_H_14_O_7_	furanonaphthoquinone	[80]
133	(2R)-6-hydroxy-7-methoxy-dehydroiso-*α*-lapachone	*Spermacoce latifolia* Aubl.	C_15_H_14_O_5_	furanonaphthoquinone	[81]
134	crassiflorone	*Diospyros crassiflora* Hiern	C_21_H_12_O_6_	furanonaphthoquinone	[82]
135	lapachol	*Tabebuia avellanedae* Lorentz ex Griseb.	C_15_H_14_O_3_	isopentenyl naphthoquinone	[83]
136	hydroxysesamone	*Sesamum indicum* L.	C_15_H_14_O_5_	isopentenyl naphthoquinone	[84]
137	2,3-epoxysesamone	*Sesamum indicum* L.	C_15_H_14_O_5_	isopentenyl naphthoquinone	[84]
138	lantalucratin D	*Lantana involucrata* L.	C_17_H_18_O_5_	isopentenyl naphthoquinone	[85]
139	lantalucratin E	*Lantana involucrata* L.	C_17_H_18_O_6_	isopentenyl naphthoquinone	[85]
140	lantalucratin F	*Lantana involucrata* L.	C_17_H_18_O_7_	isopentenyl naphthoquinone	[85]
141	butylalkannin	*Arnebia hispidissima* (Sieber ex Lehm.) A.DC.	C_20_H_22_O_6_	isopentenyl naphthoquinone	[86]
142	alkannin	*Arnebia hispidissima* (Sieber ex Lehm.) A.DC.	C_6_H_16_O_5_	isopentenyl naphthoquinone	[86]
143	rhinacanthin B	*Rhinacanthus nasutus* (L.) Kurz	C_25_H_28_O_5_	isopentenyl naphthoquinone	[59]
144	rhinacanthin C	*Rhinacanthus nasutus* (L.) Kurz	C_25_H_30_O_5_	isopentenyl naphthoquinone	[58]
145	rhinacanthin G	*Rhinacanthus nasutus* (L.) Kurz	C_25_H_30_O_6_	isopentenyl naphthoquinone	[58]
146	rhinacanthin H	*Rhinacanthus nasutus* (L.) Kurz	C_25_H_30_O_6_	isopentenyl naphthoquinone	[58]
147	rhinacanthin I	*Rhinacanthus nasutus* (L.) Kurz	C_25_H_30_O_6_	isopentenyl naphthoquinone	[58]
148	rhinacanthin J	*Rhinacanthus nasutus* (L.) Kurz	C_25_H_28_O_6_	isopentenyl naphthoquinone	[58]
149	rhinacanthin K	*Rhinacanthus nasutus* (L.) Kurz	C_25_H_32_O_7_	isopentenyl naphthoquinone	[58]
150	rhinacanthin L	*Rhinacanthus nasutus* (L.) Kurz	C_25_H_32_O_8_	isopentenyl naphthoquinone	[58]
151	cordiaquinone A	*Cordia curassavica* (Jacq.) Roem. & Schult	C_21_H_26_O_3_	isopentenyl naphthoquinone	[87]
152	chabrolonaphthoquinone A	*Nephthea chabrolii* Milne Edwards & Haime	C_27_H_32_O_4_	isopentenyl naphthoquinone	[88]
153	chabrolonaphthoquinone B	*Nephthea chabrolii* Milne Edwards & Haime	C_29_H_38_O_5_	isopentenyl naphthoquinone	[28]
154	6,8-dihydroxy-2,7-dimethoxy-3-(1,1-dimethylprop-2-enyl)-1,4-naphthoquinones	*Lysionotus pauciflorus* Maxim.	C_17_H_18_O_6_	isopentenyl naphthoquinone	[79]
155	7-hydroxy-2-*O*-methyldunniol	*Sinningia conspicua* (Seem.) Focke	C_16_H_15_O_4_	isopentenyl naphthoquinone	[89]
156	7-methoxy-2-*O*-methyldunniol	*Sinningia conspicua* (Seem.) Focke	C_17_H_17_O_4_	isopentenyl naphthoquinone	[89]
157	3,5,8-tribydroxy-6-methoxy-2-(5-oxohexa- 1,3-dienyl-1.4-naphthoquinone	*Cordyceps unilateralis* (Tul.) Petch	C_17_H_14_O_7_	isopentenyl naphthoquinone	[63]
158	rhinacanthin D	*Rhinacanthus nasutus* (L.) Kurz	C_23_H_20_O_7_	other	[58]
159	rhinacanthin M	*Rhinacanthus nasutus* (L.) Kurz	C_22_H_20_O_5_	other	[90]
160	rhinacanthin N	*Rhinacanthus nasutus* (L.) Kurz	C_27_H_24_O_7_	other	[58]
161	rhinacanthin Q	*Rhinacanthus nasutus* (L.) Kurz	C_28_H_26_O_7_	other	[58]
162	rhinacanthin U	*Rhinacanthus nasutus* (L.) Kurz	C_17_H_18_O_5_	other	[58]
163	rhinacanthin V	*Rhinacanthus nasutus* (L.) Kurz	C_25_H_22_O_6_	other	[58]
164	cordiaquinone E	*Cordia curassavica* (Jacq.) Roemer&Schultes	C_21_H_24_O_3_	other	[87]
165	cordiaquinone B	*Cordia curassavica* (Jacq.) Roemer&Schultes	C_21_H_24_O_3_	other	[87]
166	cordiaquinone K	*Cordia curassavica* (Jacq.) Roemer&Schultes	C_21_H_22_O_3_	other	[87]
167	cordiaquinone F	*Cordia curassavica* (Jacq.) Roemer&Schultes	C_26_H_30_O_5_	other	[87]
168	cordiaquinone G	*Cordia curassavica* (Jacq.) Roemer&Schultes	C_21_H_26_O_4_	other	[87]
169	cordiaquinone H	*Cordia curassavica* (Jacq.) Roemer&Schultes	C_21_H_26_O_4_	other	[87]
170	cordiaquinone J	*Cordia curassavica* (Jacq.) Roemer&Schultes	C_21_H_24_O_3_	other	[87]
171	isagarin	*Pentas longiflora*	C_15_H_12_O_4_	other	[91]
172	3-hydroxy-2-metoxy-8,8,10-trimethyl-8*H*-antracen-1,4,5-trione	*Byrsonima microphylla* A.Juss.	C_18_H_16_O_5_	other	[92]
173	3,7-dihydroxy-2-methoxy-8,8,10-trimethyl- 7,8-dihydro-6*H*-antracen-1,4,5-trione	*Byrsonima microphylla* A.Juss.	C_18_H_18_O_6_	other	[92]
174	sterekunthal A	*Stereospermum kunthianum* Cham.	C_20_H_18_O_5_	other	[62]
175	stereiqunone C	*Stereospermum kunthianum* Cham.	C_19_H_16_O_3_	other	[93]
176	sterequinone E	*Stereospermum personatum* (Hassk.) Chatterjee	C_19_H_16_O_4_	other	[93]
177	sterekunthal B	*Stereospermum personatum* (Hassk.) Chatterjee	C_20_H_18_O_4_	other	[62]
178	sterequinone B	*Stereospermum personatum* (Hassk.) Chatterjee	C_21_H_20_O_5_	other	[93]
179	3,8′-biplumbagin	*Diospyros maritima* Blume	C_22_H_14_O_6_	other	[43]
180	isozeylanone	*Plumbago zeylanica* L.	C_22_H_14_O_6_	other	[94]
181	ethylidene-3,3′-biplumbagin	*Diospyros maritima* Blume	C_24_H_18_O_6_	other	[43]
182	ethylidene-3,6′-biplumbagin	*Diospyros maritima* Blume	C_24_H_18_O_6_	other	[43]
183	ethylidene-6,6′-biplumbagin	*Diospyros maritima* Blume	C_24_H_18_O_6_	other	[95]
184	balsaminone E	*Impatiens balsamina* L.	C_22_H_16_O_5_	other	[80]
185	adenophyllone	*Heterophragma adenophyllum* Seem	C_30_H_22_O_5_	other	[96]
186	dilapachone	*Heterophragma adenophyllum* Seem	C_30_H_26_O_6_	other	[96]
187	fusarnaphthoquinone C	*Fusarium* spp.	C_29_H_26_O_11_	other	[48]
188	hygrocin A	*Streptomyces hygroscopicus* Jensen	C_28_H_31_NO_8_	other	[97]
189	hygrocin B	*Streptomyces hygroscopicus* Jensen	C_28_H_29_NO_8_	other	[97]
190	lippisidoquinone	*Lippia sidoides* Cham.	C_30_H_26_O_5_	other	[98]
191	phytonadione	*Anethum graveolens* L.	C_31_H_46_O_2_	other	[99]
192	maritinone	*Diospyros anisandra* S.F.Blake	C_22_H_14_O_6_	other	[100]

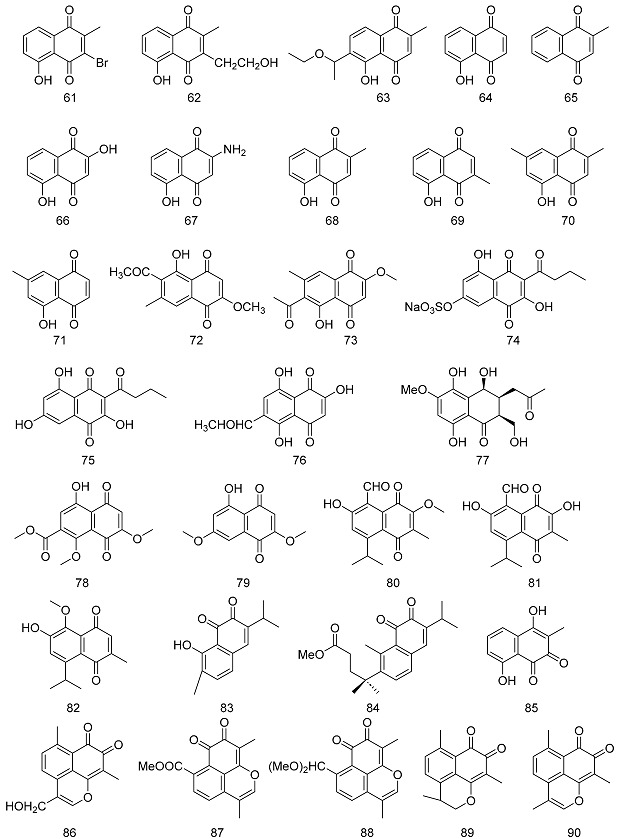

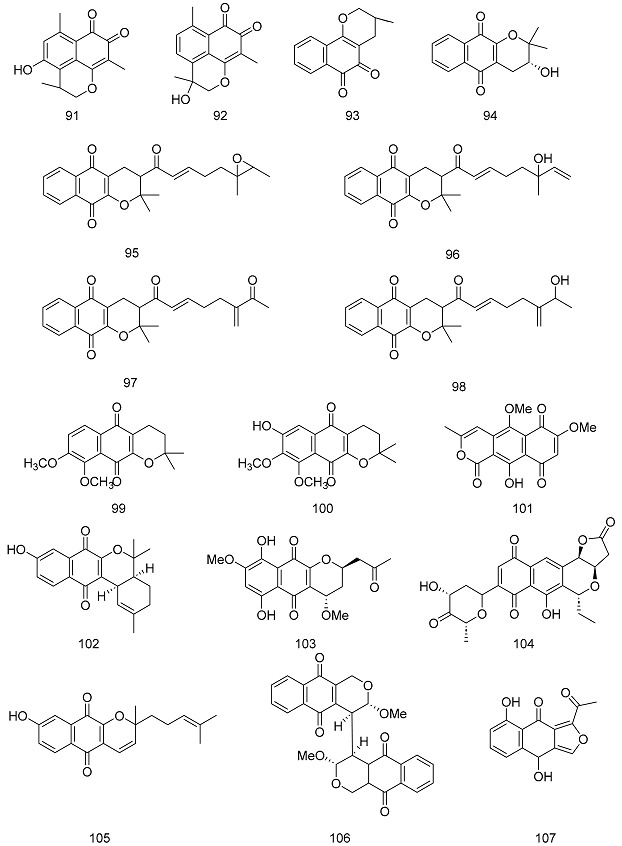

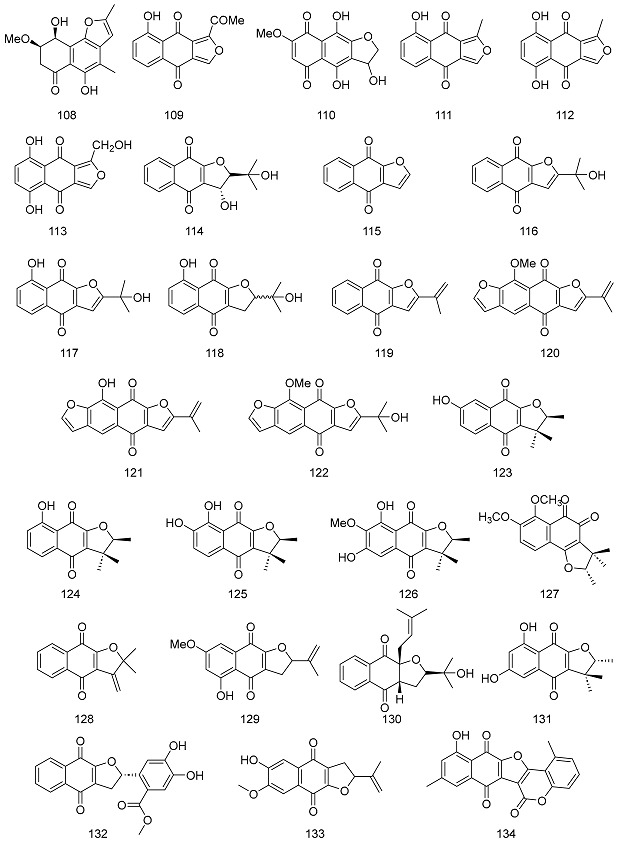

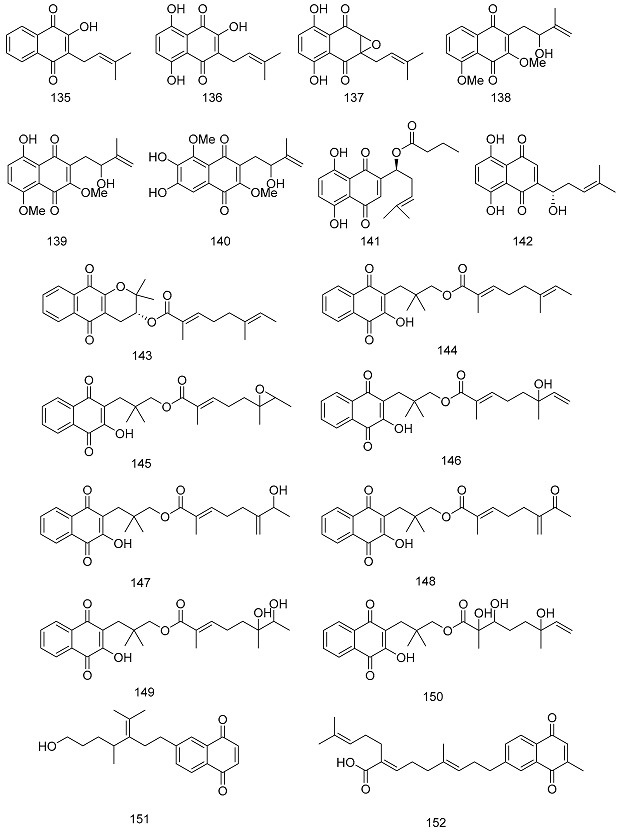

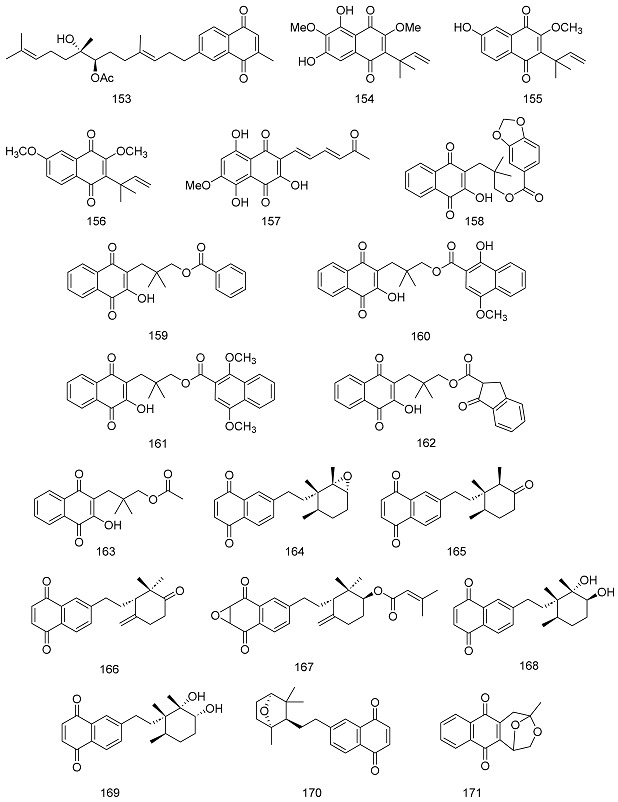

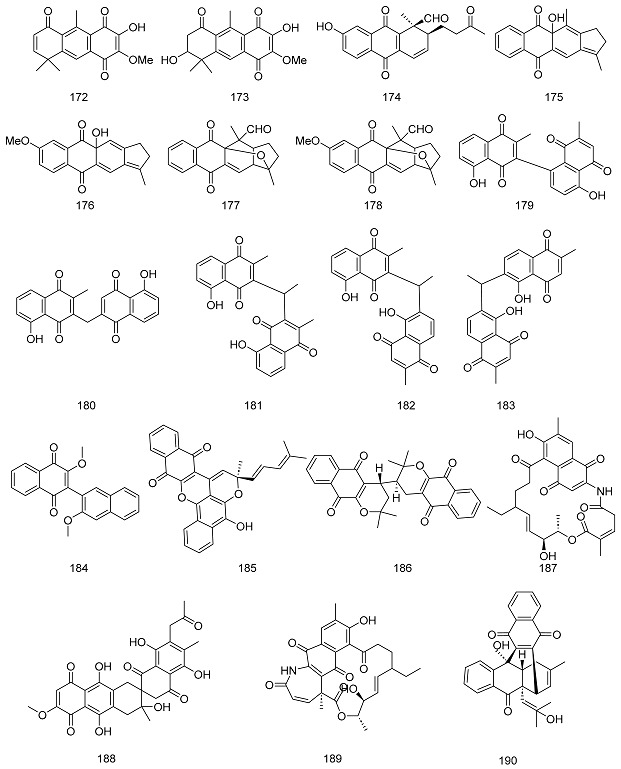

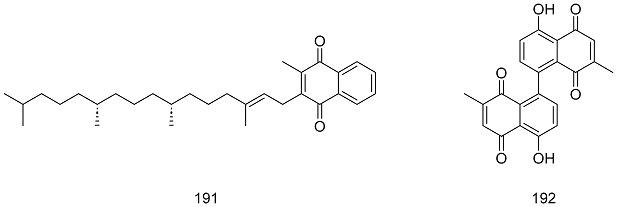















#### 2.1.3. Phenanthrenequinones

Phenanthrenequinones are an important class of natural products widely distributed in nature. These compounds are characterized by a tricyclic structure containing three rings and are classified mainly based on variations in the oxygen substitution site of the parent structure. Depending on the oxygen substitution site, phenanthrenequinones can be classified as para-oxygen substituted 1,4 phenanthrenequinone (para-phenanthrenequinone), pro-oxygen substituted 9,10 phenanthrenequinone (o-phenanthrenequinone I), and 3,4 phenanthrenequinone (o-Phenanthrenequinone II) [101].

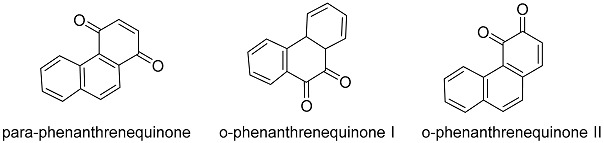



The “one-pot method has become a powerful example of resource and energy efficiency, as well as environmental sustainability. The ability to perform multiple synthetic transformations in a single reaction vessel. The pot method reduces chemical waste and makes the overall operation more environmentally friendly. Pompy Sarkar discovered the synthesis of 9,10-phenanthrenequinone by the one-pot method. In the initial step, 2-bromobenzaldehyde (**1a**) was coupled with 2-formylphenylboronic acid (**2**) under standard Pd(0) conditions. The appearance of **3a** was observed under standard Suzuki reaction conditions. The resulting product was then treated with Cu salt and TBHP. This combination leads to the formation of 9,10-phenanthrenequinone [102].

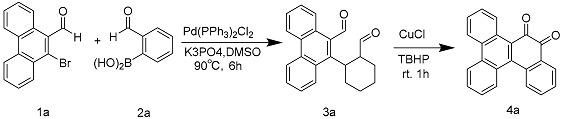



Phenanthrenequinone is mainly found in plants of *Labiatae* Juss., *Orchidaceae* Juss., and *Senecio* L., as well as in *Streptomyces* Waksman & Henrici. Among them, 11 phenanthrenequinones were isolated from *Salvia miltiorrhiza* Bunge, comprising one para-phenanthrenequinone and 10 type II o-phenanthrenequinones; six para-phenanthrenequinones were isolated from *Dendrobium nobile* Lindl.; and three phenanthrenequinones, comprising one para-phenanthrenequinone and two type II o-phenanthrenequinones, were isolated from *Salvia trijuga* Diels. Table 3 introduces the names and molecular formulas of phenanthraquinone compounds.

**Table 3:** Names and molecular formulas of phenanthraquinone compounds.

No.	Name	Resource	Formula	Classification	Ref.
193	trijuganone A	*Salvia trijuga* Diels.	C_18_H_14_O_4_	para-phenanthrenequinone	[103]
194	bauhinione	*Bauhinia variegata* L.	C_17_H_16_O_4_	para-phenanthrenequinone	[104]
195	ochrone A	*Coelogyne ochracea* Lindl.	C_13_H_12_O_4_	para-phenanthrenequinone	[105]
196	stemanthraquinone	*Stemona tuberosa* Lour.	C_16_H_14_O_4_	para-phenanthrenequinone	[106]
197	dioscoreanone	*Dioscorea membranacea* Pierre	C_16_H_12_O_5_	para-phenanthrenequinone	[107]
198	denbinobin	*Dendrobium nobile* Lindl.	C_16_H_12_O_5_	para-phenanthrenequinone	[108]
199	7-hydroxy-5,6-dimethoxy-1,4-phenanthrenequinone	*Dendrobium moniliforme* (L.) Sw.	C_16_H_12_O_5_	para-phenanthrenequinone	[109]
200	moniliformin	*Fusarium verticillioides* (Sacc.) Nirenberg	C_16_H_10_O_6_	para-phenanthrenequinone	[110]
201	phenanobiles A	*Dendrobium nobile* Lindl.	C_14_H_8_O_5_	para-phenanthrenequinone	[101]
202	phenanobiles B	*Dendrobium nobile* Lindl.	C_16_H_13_O_5_	para-phenanthrenequinone	[101]
203	phenanobiles C	*Dendrobium nobile* Lindl.	C_14_H_10_O_4_	para-phenanthrenequinone	[101]
204	6,7-dihydroxy-2-methoxy-1,4-phenanthrenedione	*Dioscorea opposita* Thunb.	C_15_H_10_O_5_	para-phenanthrenequinone	[101]
205	pyranospiranthoquinone	*Spiranthes sinensis* (Pers.) Ames	C_20_H_18_O_5_	para-phenanthrenequinone	[14]
206	ephemeranthoquinone	*Flickingeria comata* (Bl.) Hawkes.	C_15_H_12_O_4_	para-phenanthrenequinone	[111]
207	annoquinone A	*Annona montana* Macfad.	C_15_H_10_O_3_	para-phenanthrenequinone	[112]
208	danshenxinkun C	*Salvia miltiorrhiza* Bunge	C_21_H_20_O_4_	para-phenanthrenequinone	[110]
209	cypripediquinone A	*Cypripedium macranthum* Sw.	C_17_H_14_O_5_	o-phenanthrenequinone I	[111]
210	bulbophyllanthrone	*Bulbophyllum odoratissimum* (J. E. Sm.) Lindl.	C_17_H_14_O_6_	o-phenanthrenequinone I	[112]
211	Sch6 86 31	*Spiromyces* sp.	C_19_H_16_O_4_	o-phenanthrenequinone I	[14]
212	biruloquinone	*Mycosphaerella rubella* (Westend.)	C_17_H_10_O_7_	o-phenanthrenequinone I	[14]
213	danshenxinkun A	*Salvia miltiorrhiza* Bunge	C_18_H_16_O_4_	o-phenanthrenequinone II	[113]
214	danshenxinkun B	*Salvia miltiorrhiza* Bunge	C_16_H_12_O_3_	o-phenanthrenequinone II	[113]
215	danshenxinkun D	*Salvia miltiorrhiza* Bunge	C_18_H_16_O_3_	o-phenanthrenequinone II	[113]
216	cryptotanshinone	*Salvia miltiorrhiza* Bunge	C_19_H_20_O_3_	o-phenanthrenequinone II	[113]
217	tanshinone I	*Salvia miltiorrhiza* Bunge	C_18_H_12_O_3_	o-phenanthrenequinone II	[113]
218	dihydrotanshinone I	*Salvia miltiorrhiza* Bunge	C_18_H_14_O_3_	o-phenanthrenequinone II	[113]
219	tanshinone IIA	*Salvia miltiorrhiza* Bunge	C_19_H_18_O_3_	o-phenanthrenequinone II	[113]
220	hydroxytanshinone IIA	*Salvia miltiorrhiza* Bunge	C_19_H_18_O_4_	o-phenanthrenequinone II	[113]
221	tanshinone IIB	*Salvia miltiorrhiza* Bunge	C_19_H_18_O_4_	o-phenanthrenequinone II	[113]
222	miltirone	*Salvia miltiorrhiza* Bunge	C_18_H_17_O_2_	o-phenanthrenequinone II	[113]
223	trijuganone B	*Salvia trijuga* Diels.	C_18_H_16_O_3_	o-phenanthrenequinone II	[103]
224	trijuganone C	*Salvia trijuga* Diels.	C_20_H_20_O_5_	o-phenanthrenequinone II	[103]

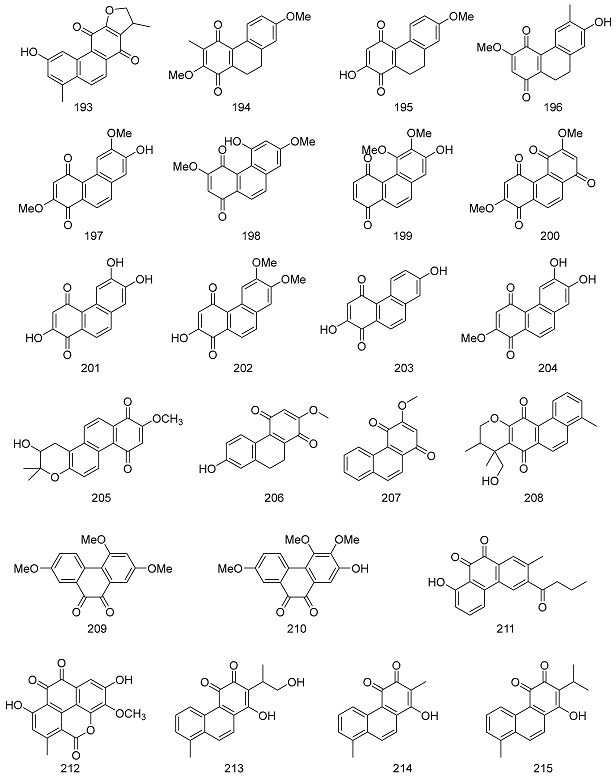

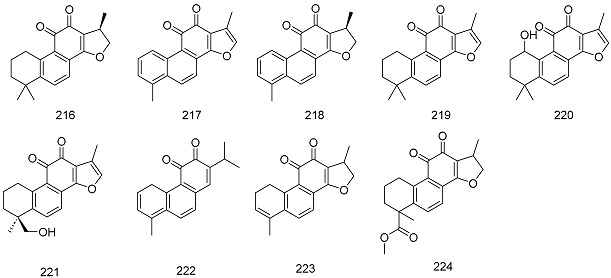





#### 2.1.4. Anthraquinones

Anthraquinones are the most abundant natural quinones [1]. Anthraquinones include anthraquinone derivatives, their reduction products, oxyanthrone or anthrone, and derivatives of their dimers. In anthraquinones, positions 1, 4, 5, and 8 are referred to as α-positions, positions 2, 3, 6, and 7 are referred to as β-positions, and positions 9 and 10 are referred to as meso-positions. The substituents of anthraquinones include methyl, hydroxymethyl, carboxyl, aldehyde, hydroxyl, and methoxy groups. Compared with benzoquinone and naphthoquinone, anthraquinone substituents contain fewer carbons, generally no more than six carbons, and the complexity and diversity of substituents are not as great as those of benzoquinone and naphthoquinone.

There are two main biosynthetic pathways for anthraquinones in medicinal plants: the polyketide pathway and the mangiferyl/pho-succinyl benzoic acid pathway [114,115,116,117]. The polyketide pathway uses acetyl coenzyme A and malonyl coenzyme A as substrates to generate anthraquinones via polyketide synthase III. The mangiferolic acid/o-succinylbenzoic acid pathway uses isobranchialic acid, α-ketoglutaric acid, and thiamine diphosphate as substrates to synthesize anthraquinones in a series of reactions catalyzed by o-succinylbenzoic acid synthase [118].

Polyketide pathway (top) and mangiferyl/phosuccinobenzoic acid pathway (bottom)

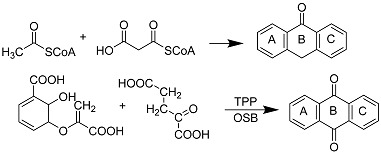



Based on the structure of the parent nucleus, anthraquinones can be categorized into two main groups: monoanthraquinones and dianthraquinones [119]. The vast majority of natural anthraquinones are found in higher plants, fungi, and lichens. Among higher plants, quinones are most abundant in the *Rubiaceae* Juss., and anthraquinones are more abundant in the *Fabaceae* Lindl. and *Rhamnaceae* Juss., *Polygonaceae* Juss., *Zygophyllaceae* R. Br., and *Liliaceae* Juss. Anthraquinones are more abundant in *Aspergillus* Micheli ex Fries and *Penicillium* spp. among molds. Twenty-one anthraquinones were found in *Pleuropterus multiflorus* (Thunb.) Nakai, including four rhodopsin-type anthraquinones, three anthraquinone glycosides, and 14 dianthrone compounds; Seventeen anthraquinones were found in *Rheum palmatum* L., containing five rhodopsin-anthraquinones, two anthraquinones oxidized, one anthrone, and seven dianthrones; thirteen anthraquinones, including three anthraquinones oxidized and nine anthraquinones, were isolated from the plant *Harungana madagascariensis* Lam. ex Poir.; ten anthraquinones were isolated and obtained from the plant *Galium sinaicum* (Delile ex Decne.) Boiss., which contains seven alizarin-type anthraquinones. Nine anthraquinones, including eight anthraquinones (including three anthraquinone glycosides) and one oxidized anthracenol, were identified in the plant *Picramnia antidesma* Sieber ex Steud.Ten anthraquinones, including three alizarin-type anthraquinones and three anthraquinone oxidizers, were found in *Rubia cordifolia* L.; Seven anthraquinones, including five rhodopsin-type anthraquinones and two rhodopsin-type anthraquinone glycosides, were found in the *Bulbine frutescens* (L.) Willd. Seven anthraquinones, including six alizarin-type anthraquinones, were found in the *Prismatomeris tetrandra* (Roxb.) K. Schum. Six anthraquinones have been found in *Stereospermum colais* (Buch.-Ham. ex Dillwyn) Mabb., and five dianthrones have been found in the *Senna alexandrina* Milll.

##### Monoanthraquinones

The vast majority of natural anthraquinones contain hydroxyl groups, and mono-anthracene-nucleated anthraquinones are usually classified into rhodopsin- and chrysophanol-types based on the substitution position of the hydroxyl group [1]. Anthraquinones with hydroxyl groups on both benzene rings belong to the rhodopsin type, such as chrysazin and chrysophorol. Anthraquinones with a hydroxyl group on one benzene ring are of the chrysin type, such as alizarin and digitolutein. Some anthraquinones also exist as glycosides. Table 4 presents the names and molecular formulas of anthraquinone compounds.

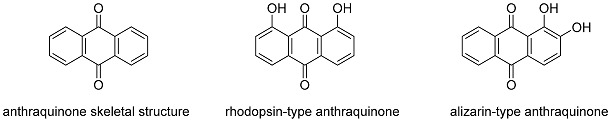



**Table 4:** Names and molecular formulas of anthraquinone compounds.

No.	Name	Resource	Formula	Classification	Ref.
225	chrysazin	*Rheum palmatum* L.	C_14_H_8_O_4_	rhodopsin-type anthraquinone	[14]
226	chrysophanol	*Rheum palmatum* L.	C_15_H_10_O_4_	rhodopsin-type anthraquinone	[14]
227	emodin	*Rheum palmatum* L.	C_15_H_10_O_5_	rhodopsin-type anthraquinone	[120]
228	isochrysophanol	*Rheum palmatum* L.	C_15_H_12_O_4_	rhodopsin-type anthraquinone	[14]
229	Rhein	*Rheum palmatum* L.	C_15_H_8_O_6_	rhodopsin-type anthraquinone	[14]
230	4-hydroxymethyl chrysazin	*Tripterygium wilfordii* Hook. f	C_15_H_12_O_5_	rhodopsin-type anthraquinone	[14]
231	1,8-dihydroxy-4-methylanthraquinone	*cyanobacterium*	C_15_H_10_O_4_	rhodopsin-type anthraquinone	[121]
232	monodictyquinone A	*Monodictys cerebriformis* G. Z. Zhao & T. Y. Zhang	C_16_H_12_O_5_	rhodopsin-type anthraquinone	[122]
233	carviolin	*Penicillium* Link ex Fr.	C_16_H_12_O_6_	rhodopsin-type anthraquinone	[123]
234	1-*O*-methylemodin	*Senna obtusifolia* (L.) H. S. Irwin & Barneby.	C_16_H_12_O_5_	rhodopsin-type anthraquinone	[124]
235	*ω*-acetylcarviolin	*Zopfiella longicaudata* (Ces.) Sacc.	C_18_H_14_O_7_	rhodopsin-type anthraquinone	[125]
236	*ω*-hydroxyemodin	*Zopfiella longicaudata* (Ces.) Sacc.	C_15_H_10_O_6_	rhodopsin-type anthraquinone	[46]
237	lunatin	*Curvularia lunata* (Wakker) Boedijn	C_15_H_10_O_6_	rhodopsin-type anthraquinone	[125]
238	ptilometric acid 6-*O*-sulfate	*Tropiometra afra macrodiscus* (Hartlaub)	C_18_H_13_NaO_10_S	rhodopsin-type anthraquinone	[46]
239	ptilometric acid	*Tropiometra afra macrodiscus* (Hartlaub)	C_18_H_14_O_7_	rhodopsin-type anthraquinone	[46]
240	cassanthraquinone A	*Cassia siamea* Lam.	C_20_H_14_O_6_	rhodopsin-type anthraquinone	[126]
241	ventilanone L	*Ventilago denticulata* Willd.	C_18_H_14_O_7_	rhodopsin-type anthraquinone	[127]
242	ventilanone M	*Ventilago denticulata* Willd.	C_18_H_16_O_6_	rhodopsin-type anthraquinone	[127]
243	1,8-dihydroxy-3-succinic acid monoethyl ester-6-methylanthraquinone	-	C_19_H_13_O_8_	rhodopsin-type anthraquinone	[128]
244	Aloe emodin	*Pleuropterus multiflorus* (Thunb.) Nakai	C_15_H_10_O_5_	rhodopsin-type anthraquinone	[44]
245	emodin methyl ether	*Pleuropterus multiflorus* (Thunb.) Nakai	C_16_H_12_O_5_	rhodopsin-type anthraquinone	[44]
246	*ω*-hydroxyemodin 8-methyl ether	*Pleuropterus multiflorus* (Thunb.) Nakai	C_16_H_12_O_6_	rhodopsin-type anthraquinone	[44]
247	emodin 8-methyl ether	*Pleuropterus multiflorus* (Thunb.) Nakai	C_16_H_12_O_5_	rhodopsin-type anthraquinone	[44]
248	vismiaquinone C	*Vismia martiana* Rchb.f.	C_21_H_20_O_5_	rhodopsin-type anthraquinone	[129]
249	asparasone A	*Aspergillus parasiticus* Speare	C_18_H_14_O_8_	rhodopsin-type anthraquinone	[130]
250	laurentiquinone A	*Vismia laurentii* De Wild.	C_22_H_20_O_7_	rhodopsin-type anthraquinone	[131]
251	laurenquinone A	*Vismia laurentii* De Wild.	C_22_H_20_O_7_	rhodopsin-type anthraquinone	[132]
252	3-*O*-(2-hydroxy-3-methylbut-3-enyl)-emodin	*Vismia guineensis* (L.) Choisy	C_20_H_18_O_6_	rhodopsin-type anthraquinone	[133]
253	3-*O*-(2-methoxy-3-methylbut-3-enyl)-emodin	*Vismia guineensis* (L.) Choisy	C_21_H_20_O_6_	rhodopsin-type anthraquinone	[133]
254	3-*O*-(E-3-hydroxymethylbut-2-enyl)-emodin	*Vismia guineensis* (L.) Choisy	C_20_H_18_O_6_	rhodopsin-type anthraquinone	[133]
255	3-*O*-(3-hydroxymethyl-4-hydroxybut-2-enyl)-emodin	*Vismia guineensis* (L.) Choisy	C_20_H_18_O_7_	rhodopsin-type anthraquinone	[133]
256	pruniflorone J	*Cratoxylum formosum* (Jack) Dyer	C_25_H_26_O_6_	rhodopsin-type anthraquinone	[134]
257	araliorhamnone A	*Araliorhamnus vaginata* H.Perrier	C_18_H_12_O_8_	rhodopsin-type anthraquinone	[135]
258	laurenquinone B	*Vismia laurentii* De Wild.	C_22_H_18_O_7_	rhodopsin-type anthraquinone	[132]
259	laurentiquinone C	*Vismia laurentii* De Wild.	C_24_H_20_O_9_	rhodopsin-type anthraquinone	[136]
260	ploiariquinone A	*Ploiarium alternifolium* (Szyszył.) Melch.	C_25_H_24_O_5_	rhodopsin-type anthraquinone	[137]
261	4′-demethylknipholone	*Bulbine capitata* Poelln.	C_23_H_16_O_8_	rhodopsin-type anthraquinone	[138]
262	knipholone	*Kniphofia foliosa* Hochst.	C_24_H_18_O_8_	rhodopsin-type anthraquinone	[139]
263	isoknipholone	*Kniphofia foliosa* Hochst.	C_24_H_18_O_8_	rhodopsin-type anthraquinone	[140]
264	knipholone-6-methyl ether	*Bulbine capitata* Poelln.	C_25_H_20_O_8_	rhodopsin-type anthraquinone	[68]
265	gaboroquinone A	*Bulbine frutescens* (L.) Willd.	C_24_H_18_O_9_	rhodopsin-type anthraquinone	[141]
266	gaboroquinone B	*Bulbine frutescens* (L.) Willd.	C_24_H_18_O_9_	rhodopsin-type anthraquinone	[141]
267	sodium *ent*-knipholone 6′-*O*-sulfate	*Bulbine frutescens* (L.) Willd.	C_24_H_17_NaO_11_S	rhodopsin-type anthraquinone	[142]
268	sodium 4′-*O*-demethylknipholone 6′-*O*-sulfate	*Bulbine frutescens* (L.) Willd.	C_23_H_15_NaO_11_S	rhodopsin-type anthraquinone	[142]
269	sodium isoknipholone 6-*O*-sulfate	*Bulbine frutescens* (L.) Willd.	C_24_H_17_NaO_11_S	rhodopsin-type anthraquinone	[142]
270	11-hydroxysulfurmycinone	*Streptomyces* sp.	C_23_H_20_O_10_	rhodopsin-type anthraquinone	[143]
271	blanchaquinone	*Streptomyces* sp.	C_22_H_20_O_7_	rhodopsin-type anthraquinone	[143]
272	brasiliquinone D	*Nocardia brasiliensis* Lindenberg & Cohn	C_28_H_29_NO_8_	rhodopsin-type anthraquinone	[144]
273	cratoxyarborequinone A	*Cratoxylum sumatranum* (Jack) Blume	C_44_H_46_O_9_	rhodopsin-type anthraquinone	[144]
274	cratoxyarborequinone B	*Cratoxylum sumatranum*(Jack) Blume	C_49_H_54_O_9_	rhodopsin-type anthraquinone	[145]
275	floribundone	*Senna septemtrionalis* (Viv.) H. S. Irwin & Barneby.	C_32_H_22_O_10_	rhodopsin-type anthraquinone	[146]
276	phaeosphenone	*Phaeosphaeria* sp.	C_30_H_26_O_10_	rhodopsin-type anthraquinone	[147]
277	R-(-)-skyrin-6-O-β-xylopyranoside	*Hypericum perforatum* L.	C_35_H_26_O_14_	rhodopsin-type anthraquinone	[148]
278	8-*O*-*β*-D-glucopyranosyl-1,1′,8′-trihydroxy- 3,3′-dimethyl-2,7′-bianthraquinone	*Eremurus chinensis* O.Fedtsch.	C_36_H_28_O_13_	rhodopsin-type anthraquinone	[149]
279	floribundiquinone A	*Berchemia polyphylla* var. *leioclada* (Hand.-Mazz.) Hand.-Mazz.	C_32_H_26_O_10_	rhodopsin-type anthraquinone	[150]
280	floribundiquinone B	*Berchemia polyphylla* var. *leioclada* (Hand.-Mazz.) Hand.-Mazz.	C_32_H_26_O_10_	rhodopsin-type anthraquinone	[150]
281	floribundiquinone C	*Berchemia polyphylla* var. *leioclada* (Hand.-Mazz.) Hand.-Mazz.	C_31_H_24_O_9_	rhodopsin-type anthraquinone	[150]
282	floribundiquinone D	*Berchemia polyphylla* var. *leioclada* (Hand.-Mazz.) Hand.-Mazz.	C_32_H_26_O_10_	rhodopsin-type anthraquinone	[150]
283	anhydrophlegmacin-9′,10′-quinone	*Cassia torosa* Cav.	C_32_H_26_O_10_	rhodopsin-type anthraquinone	[151]
284	isosengulone	*Senna multiglandulosa* (Jacq.) H.S.Irwin & Barneby.	C_32_H_22_O_10_	rhodopsin-type anthraquinone	[152]
285	icterinoidin A	*Dermocybe icterinoides* (Peck) Hesler & A.H. Sm.	C_30_H_22_O_10_	rhodopsin-type anthraquinone	[153]
286	icterinoidin B	*Dermocybe icterinoides* (Peck) Hesler & A.H. Sm.	C_30_H_22_O_10_	rhodopsin-type anthraquinone	[153]
287	febrifuquinoe	*Psorospermum febrifugum* Spach.	C_40_H_38_O_10_	rhodopsin-type anthraquinone	[154]
288	chaetomanone	*Chaetomium globosum* Kunze	C_31_H_24_O_12_	rhodopsin-type anthraquinone	[155]
289	bulbineloneside A	*Bulbinella floribunda* (Aiton) T.Durand & Schinz.	C_30_H_28_O_13_	rhodopsin-type anthraquinone	[156]
290	bulbineloneside B	*Bulbinella floribunda* (Aiton) T.Durand & Schinz.	C_28_H_24_O_12_	rhodopsin-type anthraquinone	[156]
291	bulbineloneside C	*Bulbinella floribunda* (Aiton) T.Durand & Schinz.	C_28_H_24_O_12_	rhodopsin-type anthraquinone	[156]
292	bulbineloneside D	*Bulbinella floribunda* (Aiton) T.Durand & Schinz.	C_29_H_26_O_13_	rhodopsin-type anthraquinone	[156]
293	alizarin	*Rubia cordifolial* L.	C_14_H_8_O_4_	alizarin-type anthraquinone	[14]
294	alizarin 2-methyl ether	*Rubia cordifolia* L.	C_15_H_10_O_4_	alizarin-type anthraquinone	[14]
295	digitolutein	*Ventilago goughii* Gamble	C_16_H_14_O_4_	alizarin-type anthraquinone	[14]
296	6-ethylalizarin	*Galium spurium* L.	C_15_H_12_O_4_	Alizarin-type anthraquinone	[14]
297	altersolanol A	*Stemphylium botryosum* var. *lactucum*	C_16_H_13_O_7_	alizarin-type anthraquinone	[14]
298	rubiawallin A	*Rubia wallichiana* Decne	C_16_H_12_O_5_	alizarin-type anthraquinone	[157]
299	1,4-dihydroxy-2,3-dimethoxyanthraquinone	*Hedyotis herbacea* L.	C_16_H_12_O_6_	alizarin-type anthraquinone	[158]
300	2-methoxy-1,3,6-trihydroxyanthraquinone	*Morinda citrifolia* L.	C_15_H_10_O_6_	alizarin-type anthraquinone	[159]
301	6-methylanthragallol 3-methyl ether	*Galium sinaicum* (Delile ex Decne.) Boiss.	C_16_H_12_O_5_	alizarin-type anthraquinone	[160]
302	7-methylanthragallol 1,3-dimethyl ether	*Galium sinaicum* (Delile ex Decne.) Boiss.	C_17_H_14_O_5_	alizarin-type anthraquinone	[160]
303	7-methylanthragallol 2-methyl ether	*Galium sinaicum* (Delile ex Decne.) Boiss.	C_16_H_12_O_5_	alizarin-type anthraquinone	[160]
304	7-formylanthragallol 1,3-dimethyl ether	*Galium sinaicum* (Delile ex Decne.) Boiss.	C_17_H_12_O_6_	alizarin-type anthraquinone	[160]
305	8-hydroxy-6,7-dimethoxy-2-methyl-9,10-anthraquinone	*Prismatomeris tetrandra* (Roxb.) K. Schum.	C_17_H_14_O_5_	alizarin-type anthraquinone	[161]
306	1,3-dihydroxy-5,6-dimethoxy-2-methyl-9,10-anthraquinone	*Prismatomeris tetrandra* (Roxb.) K. Schum.	C_17_H_14_O_6_	alizarin-type anthraquinone	[162]
307	3-dihydroxy-1,5,6-trimethoxy-2-methyl-9,10-anthraquinone	*Prismatomeris tetrandra* (Roxb.) K. Schum.	C_18_H_16_O_6_	alizarin-type anthraquinone	[162]
308	6-hydroxy-1, 2, 3-trimethoxy-7-methylanthracene-9, 10-dione	*Prismatomeris tetrandra* (Roxb.) K. Schum.	C_18_H_16_O_6_	alizarin-type anthraquinone	[162]
309	6-(hydroxymethyl)-1, 2,3-trimethoxyanthracene-9, 10-dione	*Prismatomeris tetrandra* (Roxb.) K. Schum.	C_18_H_16_O_6_	alizarin-type anthraquinone	[163]
310	7-hydroxy-6-(hydroxymethyl)-1, 2-dimethoxyanthracene-9,10-dione	*Prismatomeris tetrandra* (Roxb.) K. Schum.	C_17_H_14_O_6_	alizarin-type anthraquinone	[163]
311	8-hydroxyanthragallol 2,3-dimethyl ether	*Galium sinaicum* (Delile ex Decne.) Boiss.	C_16_H_12_O_6_	alizarin-type anthraquinone	[160]
312	copareolatin 5,7-dimethyl ether	*Galium sinaicum* (Delile ex Decne.) Boiss.	C_17_H_14_O_6_	alizarin-type anthraquinone	[160]
313	copareolatin 6,7-dimethyl ether	*Galium sinaicum* (Delile ex Decne.) Boiss.	C_17_H_14_O_6_	alizarin-type anthraquinone	[160]
314	5,15-dimethylmorindol	*Morinda citrifolia* L.	C_17_H_14_O_6_	alizarin-type anthraquinone	[164]
315	1,5,15-tri-*O*-methylmorindol	*Morinda citrifolia* L.	C_18_H_16_O_6_	alizarin-type anthraquinone	[165]
316	(2R)-6-hydroxy-7-methoxy-dehydroiso-α-lapachone	*Spermacoce alata* Aubl.	C_15_H_10_O_6_	alizarin-type anthraquinone	[81]
317	ventilanone N	*Ventilago denticulata* Willd.	C_16_H_12_O_6_	alizarin-type anthraquinone	[127]
318	3,4,8-trihydroxy-1-methylanthra-9,10-quinone-2-carboxylic acid methyl ester	*Eleutherine plicata* Herb.	C_17_H_12_O_7_	alizarin-type anthraquinone	[166]
319	4,8-dihydroxy-3-methoxy-1-methylanthra-9,10-quinone-2-carboxylic acid methyl ester	*Eleutherine plicata* Herb.	C_18_H_14_O_7_	alizarin-type anthraquinone	[167]
320	2-hydroxyemodin 1-methyl ether	*Senna tora* (L.) Roxb.	C_16_H_12_O_6_	alizarin-type anthraquinone	[168]
321	araliorhamnone B	*Araliorhamnus vaginata* H.Perrier	C_19_H_14_O_8_	alizarin-type anthraquinone	[135]
322	bostrycoidin	*Fusarium solani* (Mart.) Sacc.	C_15_H_11_NO_5_	alizarin-type anthraquinone	[169]
323	6-methoxylucidin*ω*-ethyl ether	*Prismatomeris tetrandra* (Roxb.) K. Schum.	C_18_H_16_O_6_	other	[161]
324	guinizarin	*Galium sinaicum* (Delile ex Decne.) Boiss.	C_14_H_8_O_4_	other	[14]
325	pachybasin	*Rheum moorcroftianum* Royle	C_15_H_10_O_3_	other	[14]
326	2-hydroxy-3-methyl-anthraquinone	*Hedyotis diffusa* Willd.	C_15_H_10_O_3_	other	[14]
327	tectoquinone	*Acatypha india* L.	C_15_H_10_O_2_	other	[14]
328	1-hydroxyanthraquinone	*Morinda officinalis* How	C_15_H_10_O_2_	other	[14]
329	2-methylol anthraquinone	*Morinda parvifolia* Bartl. ex DC.	C_15_H_10_O_3_	other	[14]
330	5-hydroxy-2-methyl-anthraquinone	*Rubia tinctorum* Linn.	C_15_H_10_O_3_	other	[14]
331	barleriaquinone I	*Barleria buxifolia* L.	C_15_H_10_O_3_	other	[14]
332	barleriaquinone II	*Barleria buxifolia* L.	C_16_H_10_O_5_	other	[14]
333	2-methylquinizarin	*Galium sinaicum* (Delile ex Decne.) Boiss.	C_15_H_12_O_4_	other	[14]
334	damnacanthol	*Damnacanthus major* Siebold & Zucc.	C_16_H_14_O_5_	other	[14]
335	ziganein	*Salvia przewalskii* Maxim.	C_15_H_10_O_4_	other	[14]
336	1-amino-2,4-dibromoanthraquinone	-	C_14_H_7_Br_2_NO_2_	other	[14]
337	munjistin methyl ester	*Salvia miltiorrhiza* Bunge	C_16_H_10_O_6_	other	[116]
338	fridamycin E	*Spiroplectammina parvula* Schwager	C_20_H_20_O_7_	other	[14]
339	soranjidiol	*Morinda elliptica* (Hook.f.) Ridl.	C_15_H_10_O_4_	other	[14]
340	*ω*-hydroxy-phomarin	*Digitalis cariensis* Boiss. ex Jaub. & Spach	C_15_H_10_O_5_	other	[14]
341	rubiawallin C	*Rubia wallichiana* Decne	C_16_H_10_O_5_	other	[157]
342	2-formyl-1-hydroxyanthraquinone	*Morinda elliptica* (Hook.f.) Ridl.	C_15_H_8_O_4_	other	[170]
343	sterequinone F	*Stereospermum colais* (Buch.-Ham. ex Dillwyn) Mabb.	C_19_H_16_O_3_	other	[170]
344	sterequinone H	*Stereospermum colais* (Buch.-Ham. ex Dillwyn) Mabb.	C_19_H_18_O_3_	other	[171]
345	1-acetoxy-3-methoxy-9,10-anthraquinone	*Rubia cordifolia* L.	C_17_H_12_O_5_	other	[172]
346	ophiohayatone C	*Ophiorrhiza hayatana* Ohwi	C_15_H_8_O_5_	other	[173]
347	munjistin-1-*O*-methyl ether	*Rhynchotechum vestitum* Wall. ex Clatke	C_16_H_10_O_6_	other	[174]
348	1,3-dimethoxy-2-methoxymethylanthraquinone	*Coussarea macrophylla* (Mart.) Müll.Arg.	C_18_H_16_O_5_	other	[175]
349	1-hydroxy-2-hydroxymethyl-3-methoxyanthraquinone	*Rubia wallichiana* Decne	C_16_H_12_O_5_	other	[157]
350	2-*n*-butoxymethyl-1,3-dihydroxyanthraquinone	*Morinda angustifolia* Roxb.	C_19_H_18_O_5_	other	[176]
351	1-methoxy-3-hydroxy-2-carbomethoxy-9,10-anthraquinone	*Saprosma scortechinii* King & Gamble	C_17_H_12_O_6_	other	[177]
352	rubiawallin B	*Rubia wallichiana* Decne	C_16_H_12_O_4_	other	[157]
353	1,7-dihydroxy-2-hydroxymethyl-9,10-anthraquinone	*Hemiboea subcapitata* Clarke	C_15_H_10_O_5_	other	[178]
354	sterequinone G	*Stereospermum colais* (Buch.-Ham. ex Dillwyn) Mabb.	C_20_H_18_O_4_	other	[171]
355	anthrakunthone	*Stereospermum kunthianum* Cham.	C_19_H_16_O_4_	other	[62]
356	3,6-dihydroxy-2-hydroxymethyl-9,10-anthraquinone	*Knoxia valerianoides* Thorel ex Pitard	C_15_H_10_O_5_	other	[179]
357	ophiohayatone A	*Ophiorrhiza hayatana* Ohwi	C_16_H_12_O_5_	other	[173]
358	pustuline	*Heterophyllaea pustulata* Hook.f.	C_16_H_12_O_4_	other	[180]
359	6-hydroxyxanthopurpurin	*Galium sinaicum* (Delile ex Decne.) Boiss.	C_14_H_8_O_5_	other	[160]
360	3-methoxycarbonyl-1,5-dihydroxyanthraquinone	*Engelhardia roxburghiana* Wall.	C_16_H_10_O_6_	other	[181]
361	1,3,6-trihydroxy-2-methoxymethyl-9,10-anthraquinone	*Saprosma scortechinii* King & Gamble	C_16_H_12_O_6_	other	[177]
362	1-methoxy-3,6-dihydroxy-2-hydroxymethyl-9,10-anthra-quinone	*Saprosma scortechinii* King & Gamble	C_16_H_12_O_6_	other	[177]
363	aloesaponarin I	*Aloe camperi* Schweinf.	C_17_H_12_O_6_	other	[182]
364	aloesaponarin I 3-methyl ether	*Aloe camperi* Schweinf.	C_18_H_14_O_6_	other	[183]
365	alatinone	*Cassia alata* L.	C_15_H_10_O_5_	other	[184]
366	przewalskinone B	*Cassia italica* Mill.	C_16_H_12_O_5_	other	[185]
367	2-Methyl-1-nitroanthraquinone	-	C_15_H_9_NO_4_	other	[186]
368	3,8-dihydroxy-6-methoxy-1-methylanthra-9,10-quinone-2-carboxylic acid methyl ester	*Gladiolus gandavensis* Van Houtte	C_18_H_14_O_7_	other	[187]
369	ventilanone O	*Ventilago denticulata* Willd.	C_16_H_12_O_6_	other	[127]
370	scorpinone	*Amorosia littoralis* Mantle & D.Hawksw. B.R.	C_16_H_13_NO_4_	other	[188]
371	1-amino-2-methylanthraquinone	-	C_15_H_11_NO_2_	other	[189]
372	dielsiquinone	*Guatteria dielsiana* R.E.Fr.	C_15_H_11_NO_4_	other	[190]
373	marcanine B	*Goniothalamus marcanii* Craib	C_16_H_13_NO_4_	other	[129]
374	marcanine C	*Goniothalamus marcanii* Craib	C_16_H_13_NO_5_	other	[123]
375	marcanine D	*Goniothalamus marcanii* Craib	C_15_H_11_NO_5_	other	[129]
376	marcanine E	*Goniothalamus marcanii* Craib	C_16_H_13_NO_5_	other	[129]
377	araliorhamnone C	*Araliorhamnus vaginata* H.Perrier	C_17_H_10_O_7_	other	[135]
378	laurentiquinone B	*Vismia laurentii* De Wild.	C_22_H_18_O_7_	other	[136]
379	sterequinone I	*Stereospermum personatum* (Hassk.) Chatterjee	C_20_H_18_O_4_	other	[171]
380	sterequinone A	*Stereospermum colais* (Buch.-Ham. ex Dillwyn) Mabb.	C_19_H_14_O_2_	other	[93]
381	sterequinone D	*Stereospermum colais* (Buch.-Ham. ex Dillwyn) Mabb.	C_20_H_16_O_3_	other	[93]
382	2-hydroxymethyl-10-hydroxy-1,4-anthraquinone	*Hedyotis herbacea* Lour.	C_15_H_10_O_4_	other	[190]
383	2,3-dimethoxy-9-hydroxy-1,4-anthraquinone	*Hedyotis herbacea* Lour.	C_16_H_12_O_5_	other	[163]
384	9,10-dimethoxy-2-methylanthra-1,4-quinone	-	C_17_H_14_O_4_	other	[191]
385	physcion	*Rheum palmatum* L.	C_16_H_12_O_5_	other	[192]
386	2-aminoanthraquinone	-	C_14_H_9_NO_2_	other	[193]
387	kengaquinone	*Harungana madagascariensis* Lam. ex Poir.	C_25_H_26_O_5_	other	[194]
388	newbouldiaquinone	*Newbouldia laevis* (P.Beauv.) Seem. ex Bureau	C_25_H_14_O_5_	other	[195]
389	newbouldiaquinone A	*Newbouldia laevis* (P.Beauv.) Seem. ex Bureau	C_25_H_14_O_6_	other	[196]
390	tectograndone	*Tectona grandis* L. f.	C_30_H_20_O_10_	other	[197]
391	(*S*)-5,5′-bisoranjidiol	*Heterophyllaea pustulata* Hook.f.	C_30_H_18_O_8_	other	[180]
392	presengulone	*Senna sophera* (L.) Roxb.	C_32_H_26_O_10_	other	[198]
393	scutianthraquinone A	*Scutia myrtina* (L.) Roxb.	C_39_H_32_O_13_	other	[199]
394	scutianthraquinone B	*Scutia myrtina* (L.) Roxb.	C_38_H_30_O_13_	other	[199]
395	scutianthraquinone C	*Scutia myrtina* (L.) Roxb.	C_34_H_24_O_12_	other	[199]
396	scutianthraquinone D	*Scutia myrtina* (L.) Roxb.	C_61_H_53_O_20_	other	[199]
397	mitoxantrone	-	C_22_H_28_N_4_O_6_	Other	[200]
398	sulfemodin 8-*O*-*β*-D-glucoside	*Rheum palmatum* L.	C_21_H_20_O_13_S	anthraquinone glycosides of rhodopsin type	[201]
399	1-methyl-8-hydroxyl-9,10-anthraquinone-3-O-β-D-glucopyranoside	*Rheum palmatum* L.	C_22_H_19_O_11_	anthraquinone glycosides of rhodopsin type	[202]
400	4′-*O*-demethylknipholone-4′-*O*-*β*-D-glucoside	*Bulbine frutescens* (L.) Willd.	C_29_H_26_O_13_	anthraquinone glycosides of rhodopsin type	[142]
401	sodium-4′-*O*-demethylknipholone-4′-*β*-D-gluc-opyranoside 6′-*O*-sulfate	*Bulbine frutescens* (L.) Willd.	C_29_H_25_NaO_16_S	anthraquinone glycosides of rhodopsin type	[142]
402	aloin	*Aloe vera* (L.) Burm.f.	C_21_H_22_O_9_	anthraquinone glycosides of rhodopsin type	[203]
403	emodin-1-O-*β*-gentiobioside	*Cassia obtusifolia*	C_27_H_30_O_15_	anthraquinone glycosides of rhodopsin type	[204]
404	knipholone-8-β-D-gentiobioside	*Bulbine narcissifolia*	C_36_H_38_O_18_	anthraquinone glycosides of rhodopsin type	[205]
405	bulbineloneside E	*Bulbinella floribunda*	C_34_H_34_O_17_	anthraquinone glycosides of rhodopsin type	[156]
406	emodin-8-*O*-*β*-D-glucopyranoside	*Pleuropterus multiflorus* (Thunb.) Nakai	C_21_H_20_O_10_	anthraquinone glucoside	[44]
407	emodin methyl ether-8-*O*-*β*-D-glucopyranoside	*Pleuropterus multiflorus* (Thunb.) Nakai	C_22_H_22_O_10_	anthraquinone glucoside	[44]
408	polygonum multiflorum ethyl	*Pleuropterus multiflorus* (Thunb.) Nakai	C_21_H_22_O_9_	anthraquinone glucoside	[44]
409	halawanone C	*Streptomycete*	C_21_H_20_O_7_	anthraquinone glucoside	[64]
410	nepalenside A	*Rumex nepalensis* Spreng.	C_21_H_22_O_11_	anthraquinone glucoside	[206]
411	nepalenside B	*Rumex nepalensis* Spreng.	C_21_H_22_O_11_	anthraquinone glucoside	[206]
412	rubiadin-3-*O*-*β*-glucoside	*Rhynchotechum vestitum* Wall. ex C. B. Clarke	C_21_H_20_O_9_	anthraquinone glucoside	[174]
413	lucidin-3-*O*-*β*-glucoside	*Rhynchotechum vestitum* Wall. ex C. B. Clarke	C_21_H_20_O_10_	anthraquinone glucoside	[174]
414	lasianthuoside A	*Lasianthus acuminatissimus* Miq.	C_22_H_22_O_10_	anthraquinone glucoside	[207]
415	lasianthuoside B	*Lasianthus acuminatissimus* Miq.	C_23_H_24_O_10_	anthraquinone glucoside	[207]
416	lasianthuoside C	*Lasianthus acuminatissimus* Miq.	C_28_H_32_O_14_	anthraquinone glucoside	[208]
417	putorinoside A	*Putoria calabrica* Pers.	C_22_H_22_O_12_	anthraquinone glucoside	[209]
418	putorinoside B	*Putoria calabrica* Pers.	C_22_H_22_O_11_	anthraquinone glucoside	[209]
419	1,3-dihydroxy-2-carbomethoxy-9,10-anthraquinone3-*O*-*β*-primeveroside	*Saprosma scortechinii* King & Gamble	C_27_H_28_O_15_	anthraquinone glucoside	[177]
420	1.3,6-trihydroxy-2-hydroxymethyl-9,10-anthraquinone 3-*O*-*β*-primeveroside	*Saprosma scortechinii* King & Gamble	C_26_H_28_O_15_	anthraquinone glucoside	[177]
421	emodin-6-*O*-*β*-D-glucopyranoside	*Reynoutria japonica* Houtt.	C_21_H_20_O_10_	anthraquinone glucoside	[210]

Anthraquinones, in a broad sense, include anthraquinone derivatives and their products with different degrees of reduction, such as oxyanthrone and anthrone. The reduction of anthraquinone in an acidic environment produces anthranol and its reciprocal isomer, anthrone. The hydroxyl derivatives of anthranol (or anthrone) often co-exist with the corresponding hydroxyl anthraquinone in plants in either the free or bound state. Table 5 presents the names and molecular formulas of oxanthrol and anthrone compounds.

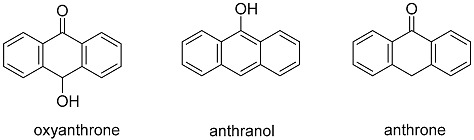



**Table 5:** Names and molecular formulas of oxanthrol and anthrone compounds.

No.	Name	Resource	Formula	Classification	Ref.
422	rubiasin A	*Rubia cordifolia* L.	C_15_H_16_O_2_	oxyanthrone	[211]
423	rubiasin B	*Rubia cordifolia* L.	C_15_H_16_O_2_	oxyanthrone	[211]
424	rubiasin C	*Rubia cordifolia* L.	C_15_H_16_O_2_	oxyanthrone	[211]
425	1-oxo-4(*S*),9-dihydroxy-8-methoxy-6-hydroxymethyl-1,2,3,4-tetrahydroanthracene	*Eremurus chinensis* O.Fedtsch.	C_16_H_16_O_5_	oxyanthrone	[149]
426	aloesaponol III-8-methyl ether	*Eremurus persicus* (Jaub. & Spach) Boiss.	C_16_H_16_O_4_	oxyanthrone	[212]
427	kenganthranol A	*Harungana madagascariensis* Lam. ex Poir.	C_30_H_36_O_5_	oxyanthrone	[194]
428	kenganthranol B	*Harungana madagascariensis* Lam. ex Poir.	C_25_H_28_O_5_	oxyanthrone	[194]
429	kenganthranol C	*Harungana madagascariensis* Lam. ex Poir.	C_26_H_30_O_6_	oxyanthrone	[194]
430	10-hydroxycascaroside C	*Rheum australe* D. Don	C_27_H_32_O_14_	oxyanthrone glycoside	[213]
431	10-hydroxycascaroside D	*Rheum australe* D. Don	C_27_H_32_O_14_	oxyanthrone glycoside	[213]
432	mayoside	*Mycobacterium microti*	C_26_H_24_O_11_	oxyanthrone glycoside	[214]
433	mayoside B	*Mycobacterium microti*	C_26_H_24_O_11_	oxyanthrone glycoside	[214]
434	mayoside C	*Picramnia teapensis* Tul.	C_33_H_34_O_16_	oxyanthrone glycoside	[215]
435	mayoside E	*Picramnia latifolia* Tul.	C_27_H_24_O_9_	oxyanthrone glycoside	[216]
436	rubanthrone A	*Rubus ulmifolius* Schott	C_17_H_14_O_10_	anthrone	[217]
437	rubanthrone B	*Rubus ulmifolius* Schott	C_17_H_16_O_9_	anthrone	[217]
438	rubanthrone C	*Rubus ulmifolius* Schott	C_16_H_12_O_10_	anthrone	[217]
439	knipholone anthrone	*Kniphofia foliosa* Hochst.	C_24_H_20_O_7_	anthrone	[218]
440	isoknipholone anthrone	*Kniphofia foliosa* Hochst.	C_24_H_20_O_7_	anthrone	[218]
441	harunganol A	*Harungana madagascariensis* Lam. ex Poir.	C_25_H_28_O_4_	anthrone	[219]
442	harunganol B	*Harungana madagascariensis* Lam. ex Poir.	C_30_H_36_O_4_	anthrone	[219]
443	harungin anthrone	*Harungana madagascariensis* Lam. ex Poir.	C_30_H_36_O_4_	anthrone	[194]
444	bazouanthrone	*Harungana madagascariensis* Lam. ex Poir.	C_30_H_36_O_5_	anthrone	[194]
445	harunmadagascarin A	*Harungana madagascariensis* Lam. ex Poir.	C_30_H_34_O_4_	anthrone	[194]
446	harunmadagascarin B	*Harungana madagascariensis* Lam. ex Poir.	C_35_H_42_O_4_	anthrone	[194]
447	harunmadagascarin C	*Harungana madagascariensis* Lam. ex Poir.	C_30_H_36_O_4_	anthrone	[220]
448	harunmadagascarin D	*Harungana madagascariensis* Lam. ex Poir.	C_30_H_36_O_5_	anthrone	[220]
449	kenganthranol D	*Harungana madagascariensis* Lam. ex Poir.	C_30_H_32_O_6_	anthrone	[220]
450	abyquinone C	*Bulbine abyssinica* A.Rich.	C_30_H_24_O_8_	anthrone	[221]
451	(*R*)-prechrysophanol	*Streptomyces* Waksman & Henrici	C_15_H_14_O_4_	anthrone	[222]
452	torosachrysone	*Dermocybe splendida* E. Horak	C_16_H_16_O_5_	anthrone	[223]
453	atrochrysone	*Aspergillus oryzae* (Ahlburg) Cohn	C_15_H_14_O_5_	anthrone	[224]
454	aloe barbendol	*Aloe vera* (L.) Burm. f.	C_15_H_14_O_4_	anthrone	[225]
455	acetyltorosachrysone	*Psorospermum glaberrimum* Hochr.	C_18_H_18_O_6_	anthrone	[226]
456	vismione H	*Psorospermum glaberrimum* Hochr.	C_22_H_24_O_6_	anthrone	[227]
457	vismione D	*Vismia orientalis* (Engl.) Byng & Christenh.	C_25_H_30_O_5_	anthrone	[228]
458	vismione L	*Psorospermum aurantiacum* Engl.	C_25_H_30_O_5_	anthrone	[229]
459	vismione M	*Psorospermum aurantiacum* Engl	C_26_H_32_O_5_	anthrone	[229]
460	asperflavin	*Microsporum* sp.	C_21_H_24_O_9_	anthrone	[230]
461	5-hydroxyaloin A	*Aloe nobilis* A.Berger	C_21_H_22_O_10_	anthrone glycoside	[231]
462	5-hydroxyaloin A 6′-*O*-acetate	*Aloe nobilis* A.Berger	C_23_H_24_O_11_	anthrone glycoside	[231]
463	picramnioside A	*Picramnia antidesma* Sieber ex Steud.	C_27_H_24_O_10_	anthrone glycoside	[232]
464	picramnioside B	*Picramnia antidesma* Sieber ex Steud.	C_22_H_22_O_10_	anthrone glycoside	[232]
465	picramnioside C	*Picramnia antidesma* Sieber ex Steud.	C_22_H_22_O_10_	anthrone glycoside	[232]
466	10-*epi*-uveoside	*Picramnia antidesma* Sieber ex Steud.	C_27_H_24_O_9_	anthrone glycoside	[233]
467	uveoside	*Picramnia antidesma* Sieber ex Steud.	C_27_H_24_O_9_	anthrone glycoside	[233]
468	microstigmin A	*Aloe microstigma* Salm-Dyck	C_30_H_28_O_13_	anthrone glycoside	[234]
469	microdontin A	*Aloe microdonta* Salm-Dyck	C_30_H_28_O_11_	anthrone glycoside	[234]
470	microdontin B	*Aloe microdonta* Salm-Dyck	C_30_H_28_O_13_	anthrone glycoside	[235]
471	cascaroside E	*Rhamnus purshiana* DC.	C_27_H_32_O_14_	anthrone glycoside	[236]
472	cascaroside F	*Rhamnus purshiana* DC.	C_27_H_32_O_14_	anthrone glycoside	[236]
473	10*R*-chrysaloin 1-*O*-*β*-D-glucopyranoside	*Rheum emodi* D. Don	C_27_H_32_O_13_	anthrone glycoside	[213]
474	isofoliosone	*Bulbine capitata* Poelln.	C_24_H_20_O_8_	anthrone glycoside	[138]
475	picramnioside D	*Picramnia teapensis* Tul.	C_26_H_24_O_10_	anthrone glycoside	[237]
476	picramnioside E	*Picramnia teapensis* Tul.	C_26_H_24_O_10_	anthrone glycoside	[237]
477	picramnioside F	*Picramnia teapensis* Tul.	C_33_H_34_O_15_	anthrone glycoside	[215]
478	picramniosdie G	*Picramnia latifolia* Tul.	C_27_H_24_O_8_	anthrone glycoside	[216]
479	picramnioside H	*Picramnia latifolia* Tul.	C_27_H_24_O_8_	anthrone glycoside	[216]
480	mayoside D	*Picramnia latifolia* Tul.	C_27_H_24_O_9_	anthrone glycoside	[216]

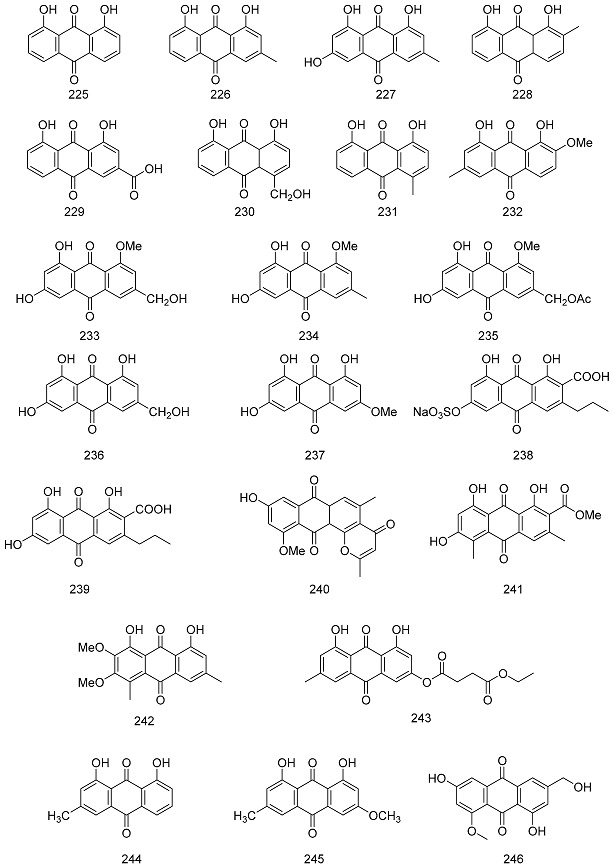

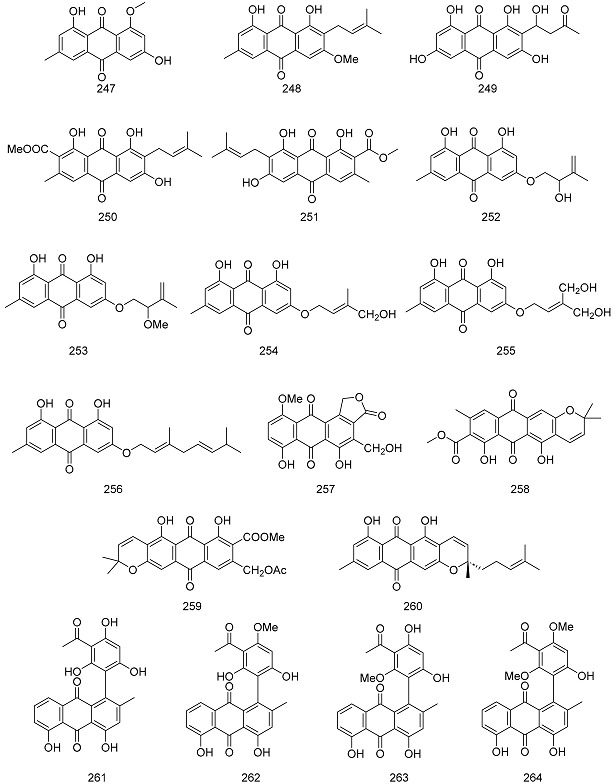

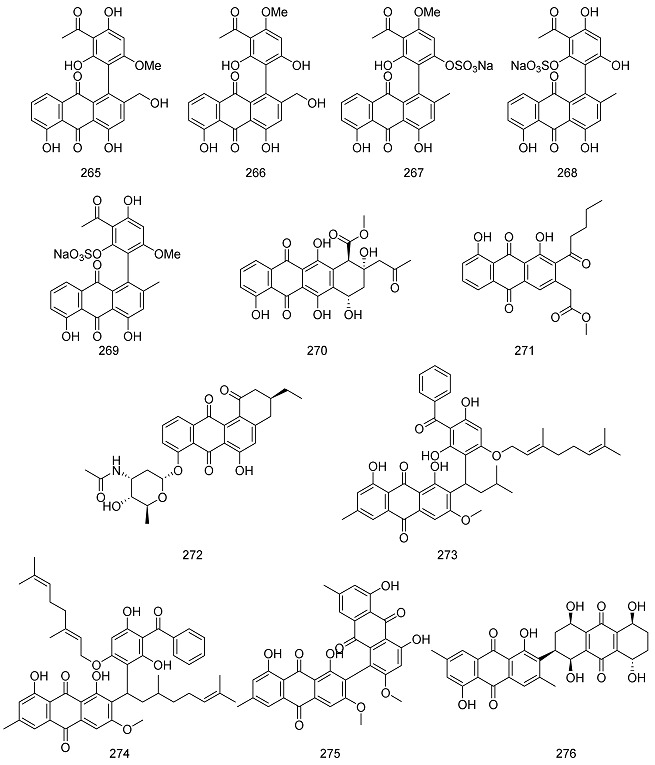

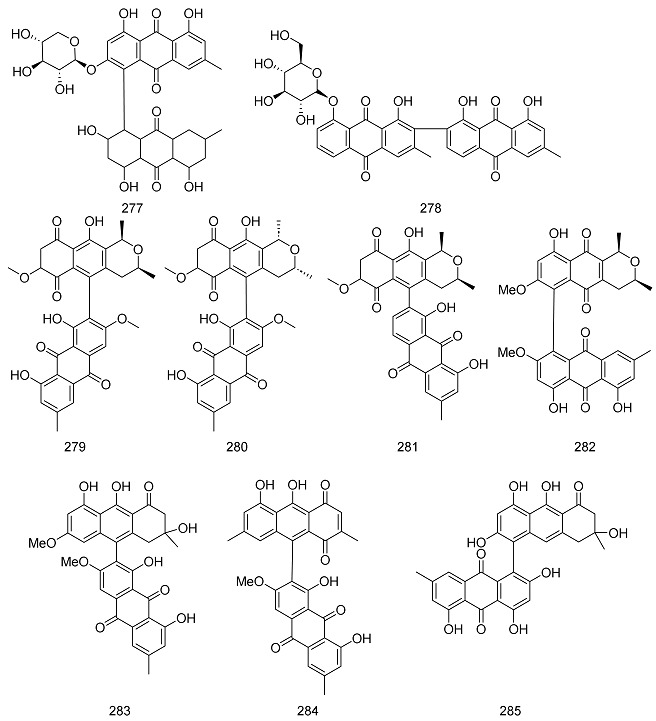

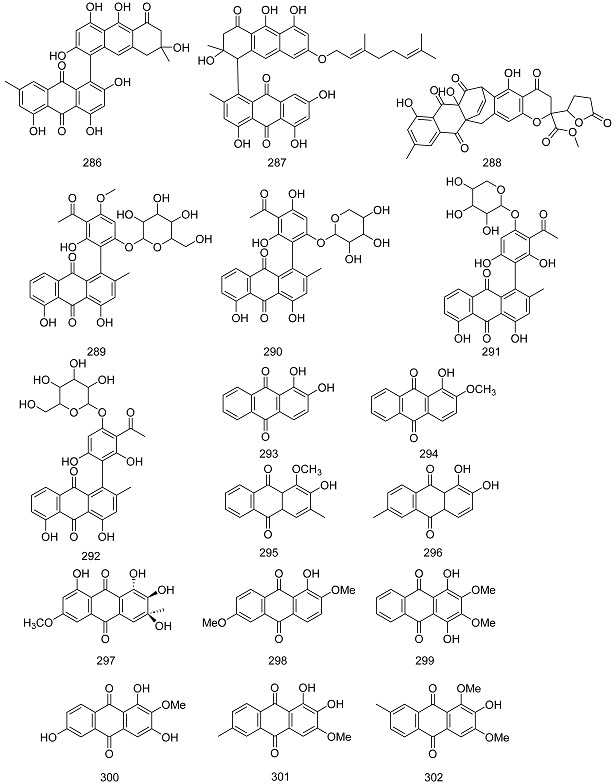

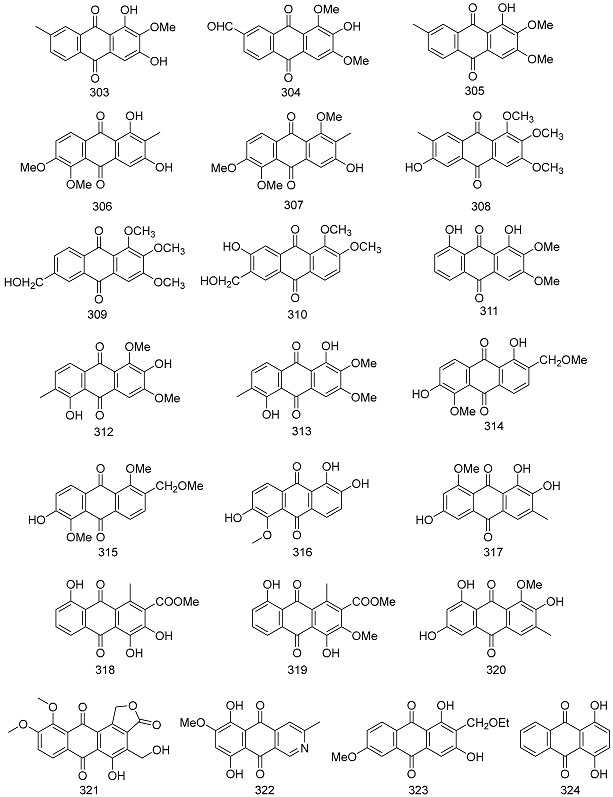

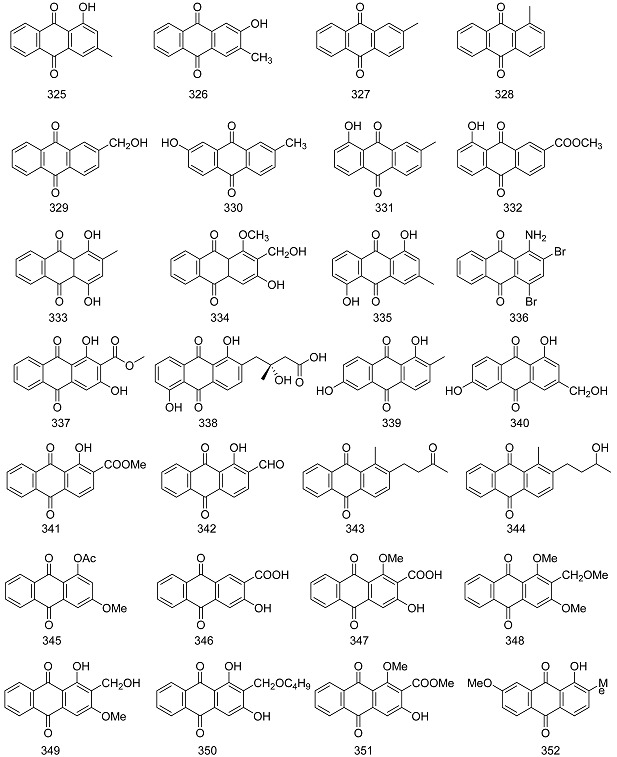

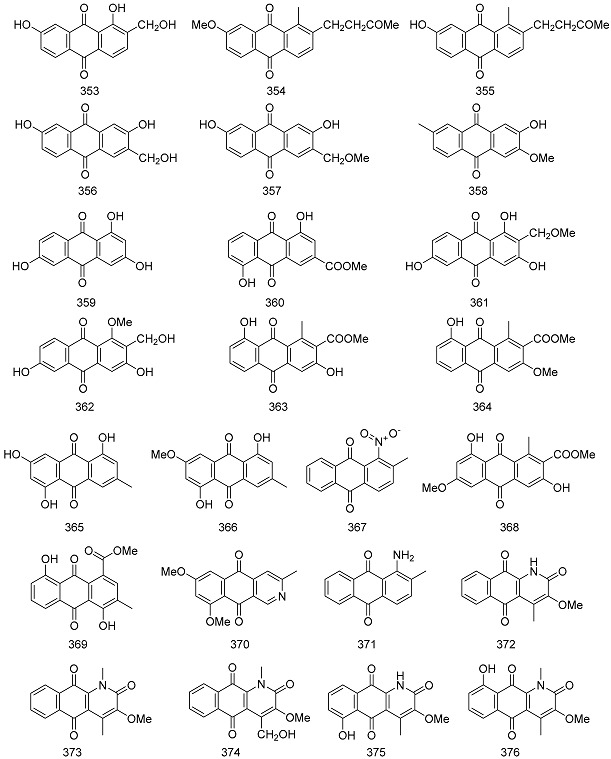

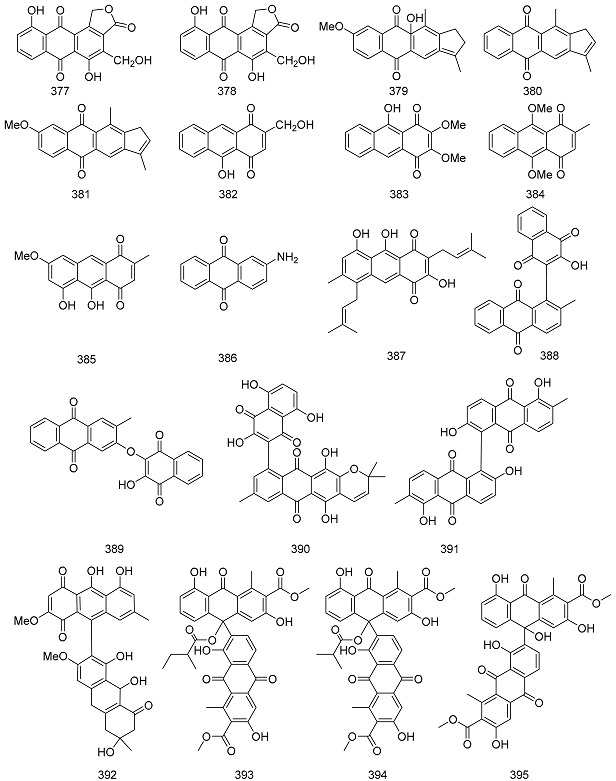

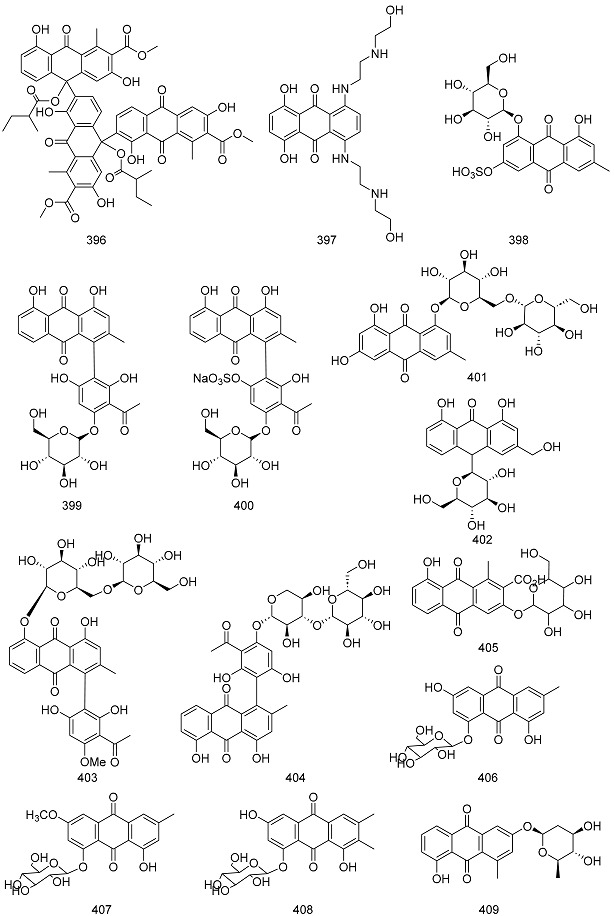

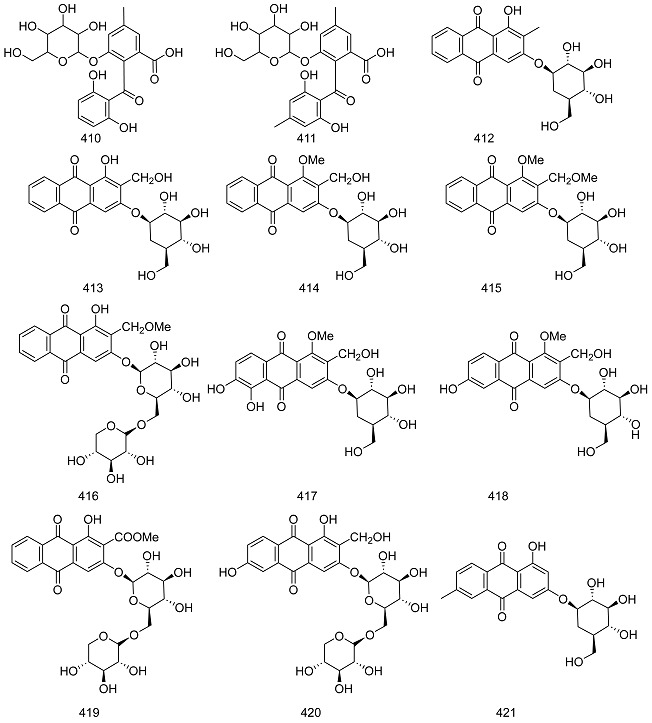

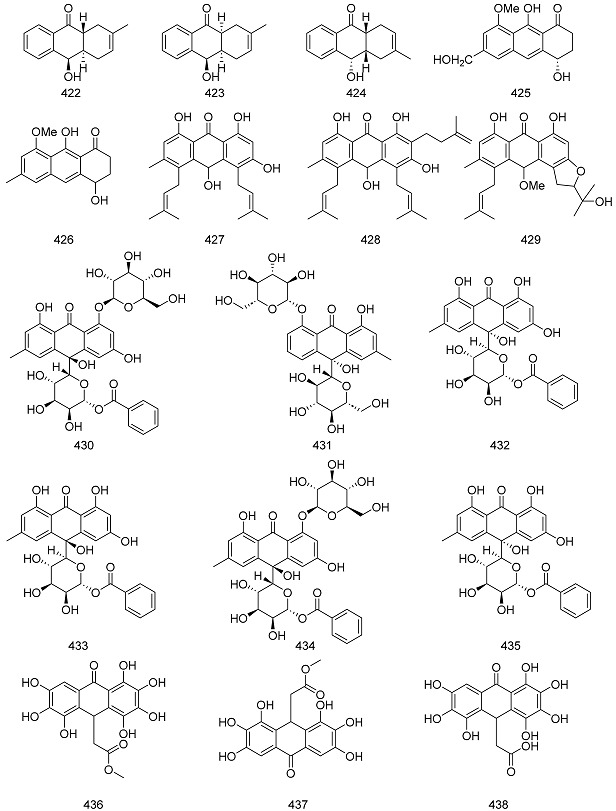

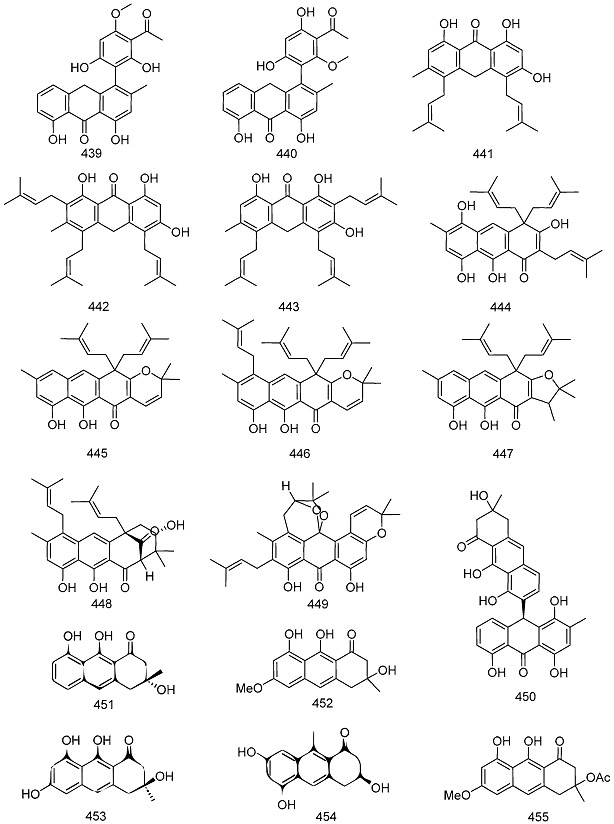

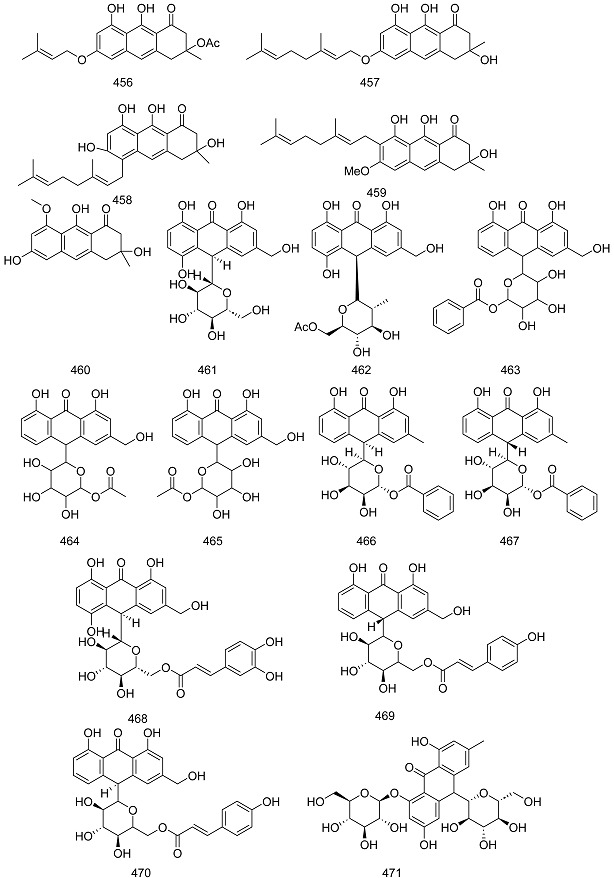

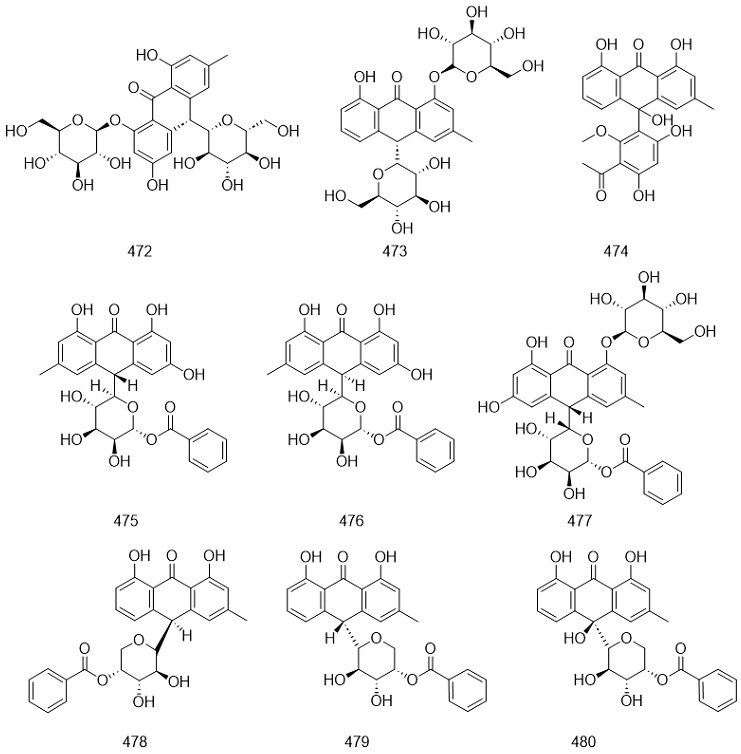































##### Dithranones

To date, about 63 species of dianthrones have been reported. These dianthrones can be classified into eight types based on their aglycone models. Type I compounds are emodin (C10→C10) emodin linked dianthrones, type II compounds are emodin (C10→C10) physcion linked dianthrones, type III are physcion (C10→C10) physcion linked dianthrones, type IV compounds are aloe-emodin (C10→C10) aloe-emodin linked dianthrones, type V compounds are rhein (C10→C10) rhein linked dianthrones, type VI compounds are rhein (C10→C10) aloe-emodin linked dianthrones, type VII compounds are chrysophanol (C10→C10) chrysophanol linked dianthrones and type VIII compounds are emodin (C10→C10) chrysophanol linked dianthrones. There are different kinds of substituent groups in these dianthrones, such as glycosylation, hydroxyl, isopentene, and malonyl groups. Table 6 introduces the names and molecular formulas of dianthrone compounds.

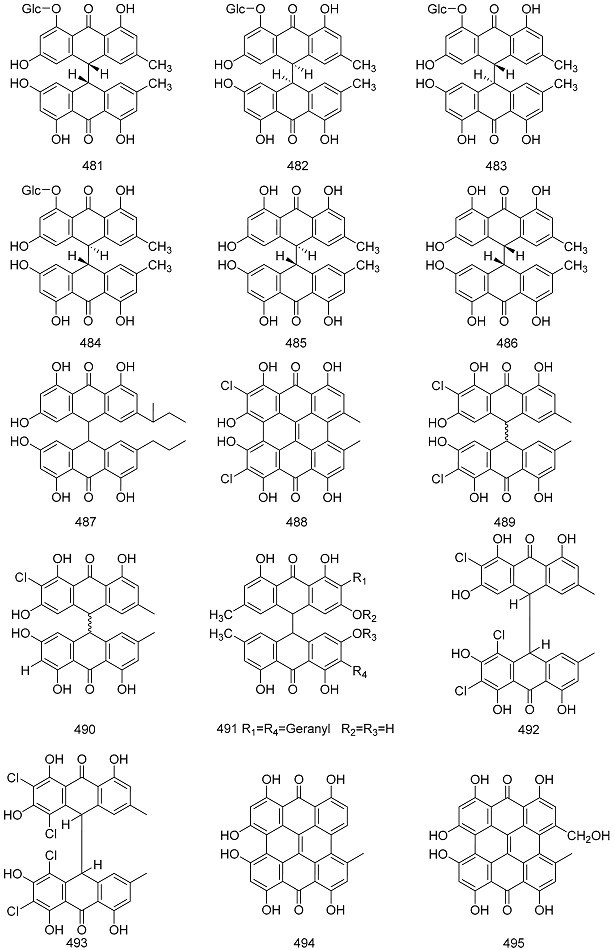

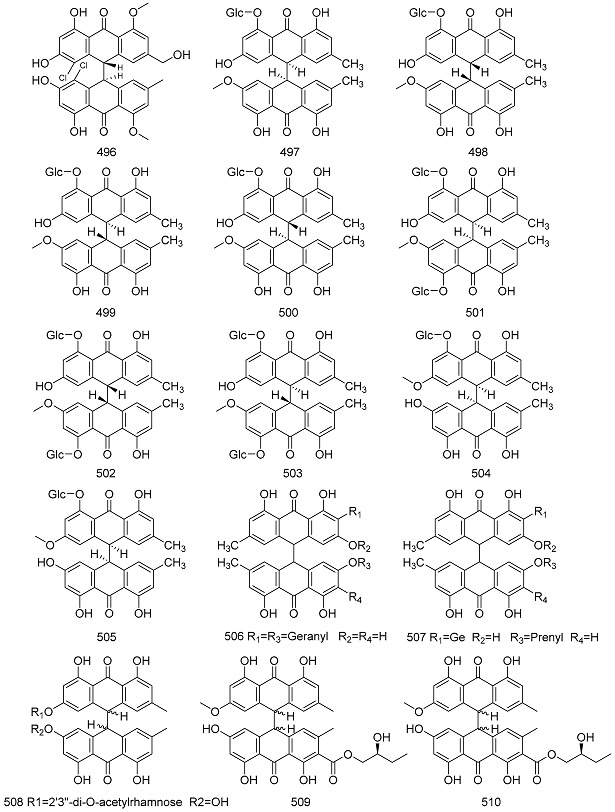

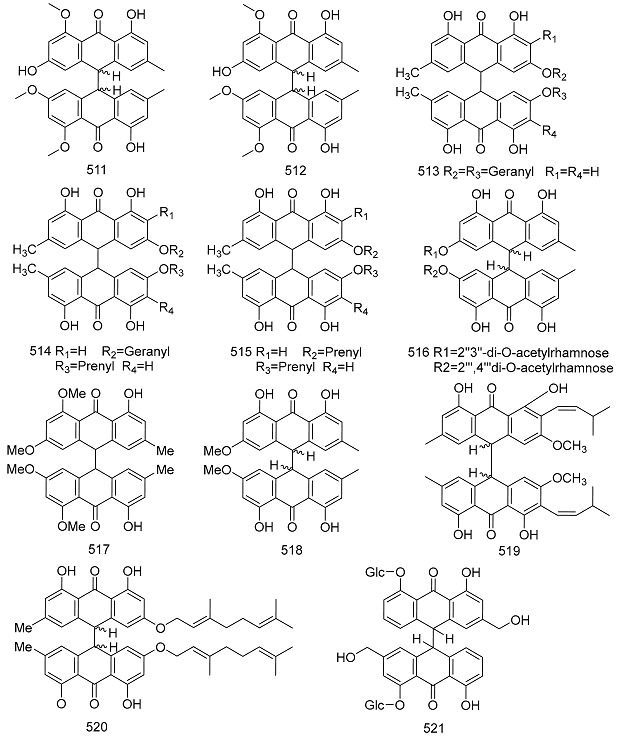

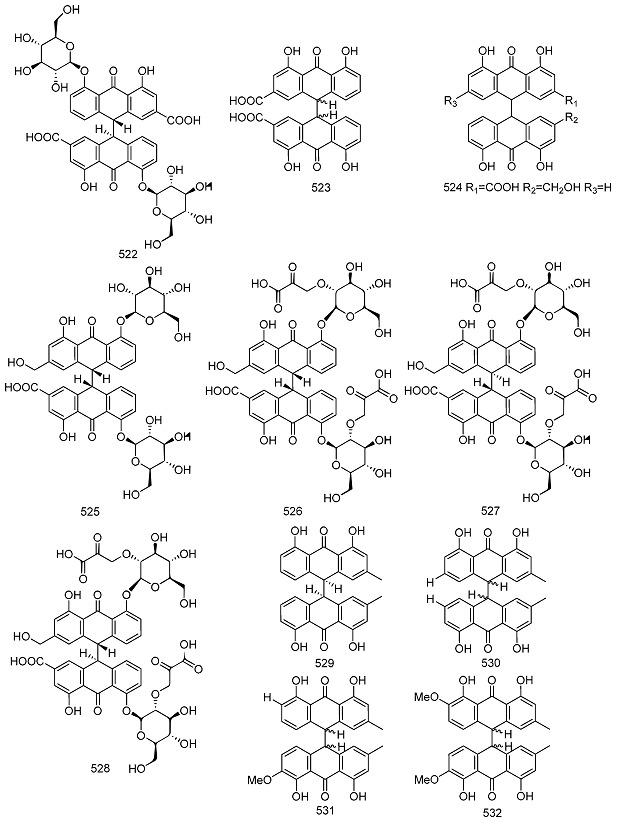

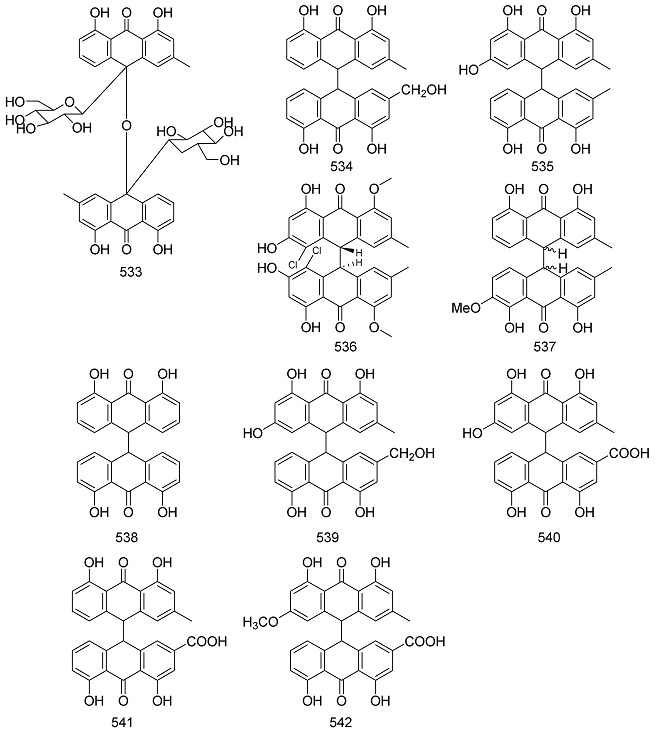











### 2.2. Extraction and Separation Methods

**Figure 4:**
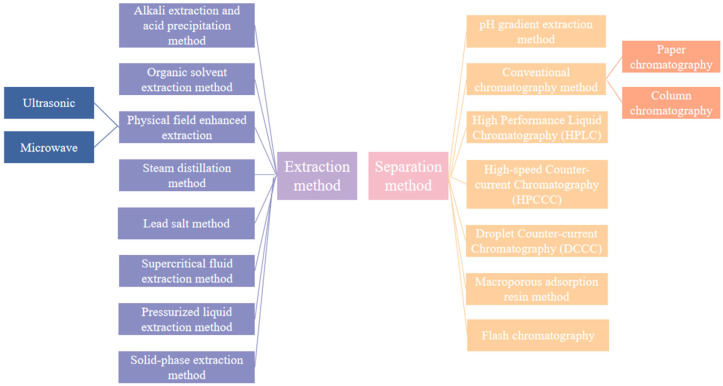
Extraction and separation methods for quinone compounds.

Quinones are the active chemical components of several traditional Chinese medicines. In nature, quinones exist in two forms: free and glycosylated. The physical and chemical properties of glycosides differ greatly, especially their polarity and solubility; therefore, their extraction and separation methods are different.Figure 4 introduces the extraction and separation methods of quinone compounds.

#### 2.2.1. Extraction

Chinese medicines often contain both anthraquinones and their glycosides. The first step in the extraction of anthraquinone glycosides is to determine whether they should be extracted simultaneously or separately. Currently, the available extraction methods include alkaline extraction and acid precipitation, organic solvent extraction, physical field-enhanced extraction, water vapor distillation, lead salt method, supercritical fluid extraction, pressurized liquid extraction, and solid-phase extraction [14].

##### Alkali Extraction and Acid Precipitation Method

The acid precipitation method is applicable to quinone compounds containing acidic groups. In the alkali extraction and acid precipitation methods, the substance to be measured is first dissolved in a suitable solvent to form a solution. Then, an appropriate amount of alkali solution was added dropwise to the solution to neutralize the acidic substance with the alkali. When the hydrogen ions in the acidic substance are completely neutralized, the resulting salt forms ions in the solution that remain dissolved. Quinone compounds with different positions and numbers of free hydroxyl groups have different degrees of acidity; therefore, they can be extracted using different concentrations of alkaline aqueous solutions. Zhang Yuebin [261] extracted cornhusk rutin by alkali extraction and acid precipitation method, and the optimal process determined by response surface method was as follows: material-liquid ratio of 1:17 (g/mL), water bath temperature of 85 °C, and water bath time of 40 min, and the extraction rate of cornhusk rutin was 6.5328%.

##### Organic Solvent Extraction Methods

The most commonly used method for extracting quinones is organic solvent extraction, and the commonly used solvents include methanol and ethyl acetate. Zhang Liangming [262] extracted the total anthraquinones from cassia seeds, and the optimal process was 70% volume fraction of ethanol, extraction time of 2.0 h, material-liquid ratio of 1:30 (g:mL), and extraction temperature of 85 °C, which resulted in a high extraction rate and a stable process. Under these conditions, the average extraction rate of total anthraquinone from cassia seed was 4.79%.

##### Physical Field Enhanced Extraction

The addition of a physical field (e.g., microwave or ultrasound) to the traditional solvent can improve the extraction effect and shorten the extraction time. Lili Cao [263] extracted anthraquinones from the rhizomes of Rubia cordifolia by an ultrasonic-assisted method. The optimal extraction conditions were an ultrasonic time of 31.29 min, solvent dosage of 13.47 mL, solvent concentration of 81.15%, and a theoretical prediction of anthraquinone extraction rate in the rhizome of Cynanchum officinale of 7.64%.

##### Steam Distillation Method

Some compounds with small relative molecular masses are volatile and can be distilled with water vapor. If the compounds are volatile and water-insoluble, they can be extracted using water vapor distillation. The water vapor distillation method is applicable to benzoquinone and naphthoquinone compounds. Du Zexiang [264] used hydrodistillation to extract quinones from the stems of Plumbago zeylanica and determined the content of plumbagin in the compounds. The results showed that the plumbagin content in the fresh and dried stems of Plumbago zeylanica was 0.0423% and 0.0420%, respectively.

##### Lead Salt Method

Lead salt precipitation is a classical method for separating certain herbal components. Since lead acetate and alkaline lead acetate can form insoluble lead salts or complex salt precipitates with a variety of herbal ingredients in aqueous and alcoholic solutions, this property can be utilized to separate the active ingredients from impurities [265].

##### Supercritical Fluid Extraction Methods

The CO_2-_supercritical fluid extraction method utilizes the properties of high density, low viscosity, and large diffusion coefficient of CO_2_ in the supercritical state to extract the active ingredients, which have the advantages of low extraction temperature, high extraction rate of the active ingredients, and short operation cycle [266]. Zhu K [267] determined the optimal extraction process for the determination of anthraquinone in Rheum officinale by CO_2_-supercritical fluid method using the orthogonal test method, the optimal extraction conditions were 40 °C maintaining 20 MPa pressure for 2 h, and using 75% ethanol as the entraining agent.

##### Solid-Phase Extraction Method

Solid-phase extraction (SPE) is a simple and convenient method for the pretreatment of samples that can effectively eliminate the interference of the sample matrix, simplify the elution conditions of liquid chromatography analysis, and shorten the analysis time. Zhao Jiangli [268] established an analytical method for the determination of hydroquinone and phenol in cosmetics by solid-phase extraction and high-performance liquid chromatography, and the detected concentration andquantitative concentration can meet the technical requirements of the «Cosmetic Safety Code», which can be used for the determination of hydroquinone and phenol in cosmetics with complex matrices.

##### Pressurized Liquid Extraction Method

The pressurized liquid extraction method uses a conventional solvent to extract solid or semi-solid samples under relatively high temperature and pressure [269]. Ong and Soon [270] employed pressurized liquid extraction (PLE) to extract thermally unstable components, such as tanshinone I and tanshinone IIA, from Salvia miltiorrhiza Bunge. PLE was carried out dynamically under the following conditions: a flow rate of 1 mL/min, temperature of 95–140 °C, applied pressure of 10–20 bar, and extraction times of 20 and 40 min. The extraction efficiency of PLE is higher than that of other methods.

#### 2.2.2. Separation

##### pH Gradient Extraction Method

**Figure 5:**
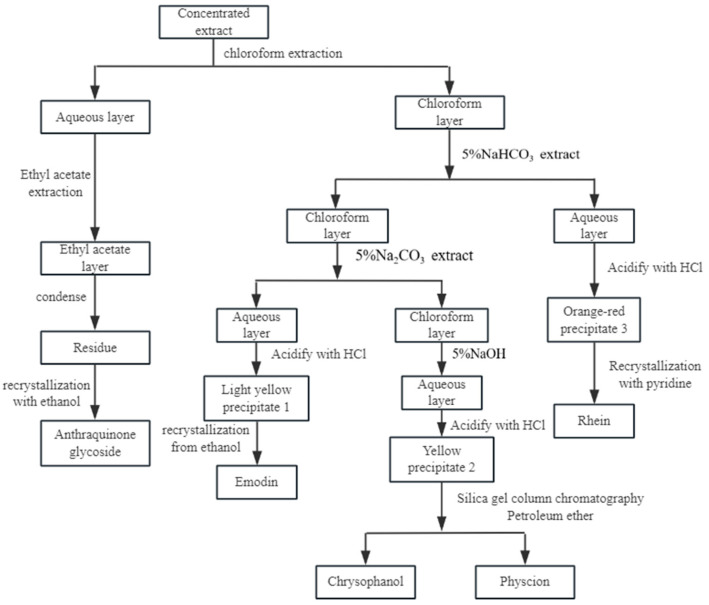
Flow chart of the separation of anthraquinone compounds from pomegranate peels.

pH gradient extraction is a traditional method for separating quinones. Quinones contain free hydroxyl groups at different locations and numbers, with different acidic strengths, and different quinones can be selectively extracted using different concentrations of alkaline aqueous solutions [271]. He Ying [272] determined the anthraquinones in the browning products of pomegranate pericarp and used pH gradient extraction for separation and column chromatography purification to obtain four anthraquinones, which were identified as rhubarb phenol, rhubarb, rhubarb acid, and rhubarb methyl ether, and the optimal process conditions were ethanol concentration of 75%, ethanol dosage of 90 mL, extraction time of 25 min, and extraction temperature of 25 °C. The extracts were extracted at 25 °C, and the extracts were extracted at 25 min.Figure 5 introduces the flow chart for the separation of anthraquinone compounds from pomegranate peels.

##### Chromatographic Methods

The most commonly used method for separating quinones is chromatography, which is particularly effective for separating quinones with free phenolic hydroxyl groups, especially anthraquinones. Conventional chromatographic methods include paper chromatography and column chromatography. An increasing number of new techniques have been applied to the separation of quinone compounds, such as high-performance liquid chromatography, high-performance countercurrent chromatography, droplet countercurrent chromatography, large-pore adsorbent resin, and flash column chromatography. High-performance liquid chromatography (HPLC) is a great complement to traditional chromatography, and with the continuous development of technology, HPLC has been greatly improved, and its operation and data processing are more automated. Chromatographic columns are packed with an ever-increasing variety of materials that can separate substances under normal-phase, reversed-phase, and even chiral conditions.HPLC instruments can be connected to a wide variety of monitors and are increasingly used in the separation of quinones. Jun Huang [273] established a high-performance liquid chromatographic assay for the separation of lawsone, which was sensitive, rapid, and simple, and was corroborated by high-performance liquid chromatography-tandem mass spectrometry to ensure accurate results. High-speed countercurrent chromatography (HSCCC) is a continuous liquid-liquid chromatographic technique that does not require solid-phase carriers. Tian, G [274] used multidimensional high-performance countercurrent chromatography to obtain four major components, tanshinone IIA, tanshinone I, dihydrotanshinone I, and cryptotanshinone II, with purities above 95%. Droplet countercurrent chromatography (DCCC) separates compounds based on differences in partitioning between two immiscible liquid phases. This method requires the system to be separated into two phases in a short period and form droplets efficiently [275].

##### Macroporous Adsorption Resin Method

Macroporous adsorption resin separation technology is a process of extraction and refinement that uses special adsorbents to selectively adsorb the active ingredients and remove the ineffective ingredients from the compound decoction of traditional Chinese medicine [276]. Zhenkang Lu [277] used macroporous adsorbent resin for the separation and purification of Juglans cyan bark pigment, and the dynamic adsorption and desorption experiments showed that the D-101 macroporous adsorbent resin was the most effective for the separation and purification of Juglans cyan bark pigment. The optimum conditions for adsorption were an initial concentration of 1.5 mg/mL, a flow rate of 0.5 mL/min, a pH of 3, and a volume of 50 mL of sample solution. The optimum conditions for desorption were an elution flow rate of 1.5 mL/min, ethanol concentration of 90% in the eluate, and elution pH of 4.

### 2.3. Structural Identification Methods

Common methods for the structural identification of benzoquinone include ultraviolet absorption spectroscopy, infrared absorption spectroscopy, nuclear magnetic resonance spectroscopy, and mass spectrometry.

#### 2.3.1. Benzoquinones

Benzoquinones exist in a long conjugated system, and in the UV absorption spectrum, the molecules can show long absorption peaks in both the near-UV and visible regions. The three main absorption bands of benzoquinone are: ca. 240 nm (strong absorption); ca. 285 nm (medium to strong absorption); and ca. 400 nm (weak absorption). Benzoquinones are most characterized in the infrared spectra by the telescopic vibrational absorption peaks of carbonyl, hydroxyl, and double bonds at 1675–1653 cm^−1^ (ν_C=O_), 3600–3140 cm^−1^ (ν_OH_), 1640–1200 cm^−1^ (ν_C=C_). The number of absorption peaks and the wavenumber of the carbonyl group of the benzoquinone compound are closely related to the substituents on benzoquinone. When there is a hydroxyl substitution in the molecule, the hydrogen bonding between the carbonyl group and the hydroxyl group will cause a significant decrease in the wavenumber of the carbonyl absorption peak. If the molecular structure is symmetrical after the substitution of the substituent group, the compound is the same as unsubstituted benzoquinone, and there is only one base absorption peak in the infrared absorption spectrum. In the NMR hydrogen spectrum, the chemical shift of the unsubstituted benzoquinone ring proton is δH 6.72(s); when there is a substitution of an electron-donating group on the ring, it causes the chemical shifts of the other protons to be shifted to the higher field. In the NMR carbon spectra, the chemical shift of the unsubstituted benzoquinone carbonyl carbon is around δC 187, and substitution of the substituents around the benzoquinone carbonyl group induces a shift in the chemical shift of the carbonyl carbon. In the mass spectrum, the chemical shift of the carbonyl carbon is shifted to a higher field when the electron-donating group is substituted. In the mass spectrum, the molecular ion peak of unsubstituted benzoquinone is *m*/*z* 108, and cleavage fragments of *m*/*z* 82, *m*/*z* 80, and *m*/*z* 54 appear in its mass spectrum. A fragmentation ion peak (*m*/*z* 52) with two consecutive CO removals was present in the mass spectrum of benzoquinone. For substituted benzoquinones, this cleavage pattern provides an important basis for deducing the type of substituent.

#### 2.3.2. Naphthoquinones

The UV absorption of naphthoquinone mainly originates from two parts of the structure: the naphthalene-like and quinone-like structures. The naphthalene structure has three main absorption bands at 245, 251, and 335 nm, while the quinone structure has a main absorption band at 257 nm [1]. When OH^−^, OCH_3_^−^, and other electron-donating groups are substituted in the molecule, the corresponding absorption bands are redshifted. The characteristic absorption peaks in the IR pattern of naphthoquinone remained in the carbonyl stretching vibration absorption peak from 1675 to 1653 cm^−1^ and the backbone vibration absorption peak between 1635 and 1648 cm^−1^ of the aromatic ring. In the NMR hydrogen spectra, when there is no substituent on the naphthoquinone (1.4-naphthoquinone) ring, the chemical shift of the ring proton is δH 6.95. In NMR carbon spectra, when there is an electron-donating substituent on the quinone ring, the quinone ring proton is shifted to the high field, and the degree of shift is related to the magnitude of the electron-donating effect [278]. When there is an electron-donating substituent on the quinone ring, such as C3 substituted with -OH or -OR, the chemical shift of C-3 is shifted to the low field by about 20 ppm, and that of C-2 is shifted to the high field by 30 ppm. When the C2 substituent is R, the C-2 signal shifts to the low field by about 10, and the C3 signal shifts to the high field by about 8. The extent of the shift of C2 to the low field increased with increasing R. The C2 substituent is a substituent of the C2 position in quinone rings.

#### 2.3.3. Phenanthrenequinones

Phenanthrenequinone, although structurally classified as a phenanthrenequinone, is biosynthetically classified as a diterpene quinone based on the structures of other coexisting congeners [11]. The vast majority of diterpene quinones have a rosinane or rearranged rosinane-type skeleton and include many pro-quinone types. Most quinone carbonyls in this group are present on the C-ring of the rosinane diterpenes, with 1,4-p-quinone and, in a few cases, also the o-type, usually with an isopropyl unit on the C-ring. The presence of this structural unit can be judged mainly by the chemical shifts of the protons and the shape of the peaks on ′H NMR. Most of the quinone carbonyls in this group are present on the C-ring of the rosinane diterpenes, with 1,4-p-quinone and, in a few cases, also the o-type, usually with an isopropyl unit on the C-ring, and the presence of this structural unit can be judged mainly by the chemical shifts of the protons and the shape of the peaks on ^1^H NMR. Generally, the chemical shifts of 16-CH3 and 17-CH3 are around 1.10, each appearing as a double peak. The C-15 hypomethyl proton appeared around 3.00 and showed a heptagonal peak due to coupled cleavage with both methyl protons. The ^13^C NMR chemical shift values of the carbonyl group are mainly derived from the presence or absence of hydroxyl groups in the neighboring environment, and the chemical shift of the carbonyl group with hydrogen bonding is shifted to a lower field. Methyl, hydroxyl, acetyl, and a third carbonyl group are also often present in the diterpene skeleton structure, and the substitution positions of these groups are usually based on two-dimensional mapping. The positions of these substituents are usually determined by a comprehensive analysis of the ^1^H COSY, HMQC, and HMBC spectra. In addition, the diterpene skeleton is often broken in this type of structure, and the identification of its structure is also mainly based on the analysis of NMR data. Where conditions permitted, confirmation was made by X-ray single-crystal diffraction data analysis [14].

#### 2.3.4. Anthraquinones

In the UV absorption spectrum, anthraquinone has four main absorption bands caused by the benzene-like and quinone-like structures, with four absorption peaks at 252, 325, 272, and 405 nm. Most natural anthraquinones have hydroxyl substitutions, and the UV absorption spectra of hydroxy anthraquinones have five main absorption peaks: the I absorption peak is around 230 nm; the II absorption peak is 240–260 nm (caused by the benzene-like structure); the III absorption peak is 262–295 nm (caused by the quinone-like structure); the IV absorption peak is 305–389 nm (caused by the benzene-like structure); and the V absorption peak is greater than 400 nm (caused by C=O in the quinone-like structure) [1]. The information provided by the UV-Vis spectra of anthraquinones is of some use for structural speculation; however, because of the plethora of exceptions, UV-Vis spectral data are usually used only as circumstantial evidence for structural analysis. The IR absorption spectra of hydroxyanthraquinone are characterized by carbonyl stretching vibrational absorption near 1670 cm^−1^. Hydroxyl stretching vibrational absorption in the 3600–3150 cm^−1^ interval and benzene ring backbone vibrational absorption in the 1600–1480 cm^−1^ interval [279].In ^1^H NMR, the NMR signals of the aryl hydrogens of the anthraquinone parent nucleus can be divided into two categories: α-aryl hydrogens are in the negatively shielded region of C=O, which are more affected by the carbonyl group, and the resonance occurs in the lower magnetic field region, with the peak centered around δ8.07 [280]; β-aryl hydrogens are less affected by the carbonyl group, and the resonance occurs in the higher magnetic field region, with the peak centered around δ6.67 [271]. ^13^C NMR plays an important role in the identification of quinones. ^13^C NMR is important for the identification of quinones. The carbon atoms of the quinone parent nucleus can be classified into four groups, and the chemical shift values of these carbons in unsubstituted anthraquinones are as follows: α-C 126.6, β-C 134.3, carbonyl carbon 182.5, and quaternary carbon 132.9. When there is a hydroxyl substitution at the α-position, the chemical shift of the carbonyl carbon is shifted to the lower field to about 187 [14].

## 3. Progress in Pharmacological Activity Research

Quinones are abundant in nature, and their pharmacological activities, including immunomodulatory, antitumor, anti-inflammatory, antibacterial, antioxidant, and laxative effects, have received widespread attention.

### 3.1. Immunomodulatory Effects

Quinones exert multiple regulatory effects on the immune system. At the level of immune cells, it can activate macrophages to enhance phagocytosis, regulate their polarization, affect the differentiation and cytotoxicity of T-lymphocyte subpopulations, and regulate the activation and proliferation of B-lymphocytes and antibody secretion. Shen Jie established an SLE model and tested the parameters of lymph node size, spleen index, kidney index, Th cell subpopulation, and B cell activation index in mice. After Embelin treatment, the Th1/Th2 and Treg/Th17 ratios in the lymph nodes and spleens of SLE mice were significantly elevated. Moreover, the concentrations of dsDNA, ssDNA, and IgG in the serum of mice were significantly decreased. It was concluded that embelin exerts a therapeutic effect on SLE mice by regulating the balance of Th cell subpopulations and inhibiting the activation of Th and B cells, demonstrating that letterbox quinone has immunomodulatory and therapeutic effects on SLE [281].

### 3.2. Anti-Tumor Activity

Quinones exhibit anti-tumor effects. On the one hand, quinones can induce apoptosis in cancer cells by activating the endogenous apoptotic pathway. On the other hand, quinones can interfere with the cell cycle of tumor cells, causing them to stagnate at a certain stage and inhibiting the proliferation of tumor cells. In addition, quinones can inhibit tumor angiogenesis and reduce nutrient supply to tumors. Moreover, it can enhance the immune function of the body and activate immune cells to recognize and kill cancer cells, thus playing a multi-faceted positive role in the anti-tumor process. Common antitumor components include embelin [282], emodin [283], chrysophanol [284], tanshinone IIA [285], juglone [286], plumbagin [287], aloe-emodin [288], dioscoreanone [289], and denbinobin [290]. Table 7 introduces the anti-proliferative effects of quinone compounds on cells.

Avci, H [286] used MTT to determine the cytotoxic effect of juglone. Treatment of BxPC-3 human pancreatic cancer cells with different concentrations of juglone reduced the expression of MMP-2 and -9 genes in a dose-dependent manner, and VEGF induced a significant reduction in the level of expression of Phactr-1 gene, indicating that huperzine has an anti-metastatic effect on human pancreatic cancer cells. Zhang utilized the thiazolyl blue reduction method (MMT) to detect the antiproliferative effect of Dendrobium officinale phenanthrenequinone on human ovarian cancer cells HO-8910PM, while the Transwell assay was used to detect changes in the metastatic ability of the cells. The expression of apoptosis- and metastasis-related genes and protein levels in HO-8910PM cells was detected using reverse transcription-polymerase chain reaction and protein blotting. The results of the MTT assay showed that the proliferation inhibitory effect of dendrobium phenanthrenequinone at 3 μmol/L and 10 μmol/L on ovarian cancer cells was significant, and dendrobium phenanthrenequinone inhibited the proliferation and metastasis of ovarian cancer cells by upregulating the expression of CASP3, CASP9, and CAV1, and downregulating the expression of SOX2. The experimental results demonstrated that dendrobium phenanthrenequinone has anti-invasive and metastatic therapeutic effects on human ovarian cancer cells [291]. Yang suggested that rhodopsin inhibited SREBP1-dependent and SREBP1-non-dependent cell proliferation and led to caspase-dependent and caspase-non-dependent induction of endogenous apoptosis in HCC [292]. The IC50 value of rhodopsin in L02 cells was 36.69 μg/L [293]. The toxicity of rhodopsin on normal human cells (IC50 values ranging from 92.59 to 185.18 μmol/L) was slightly lower than the IC50 values of rhodopsin on cancer cells (10 to 80 μmol/L).

### 3.3. Antioxidant Activity

Anthraquinones possess antioxidant effects and play a positive role in protecting the body against oxidative stress damage. Quinones with antioxidant effects include idebenone [294], plumbagin [295], juglone [296], alkannin [297], tanshinone I [298], tanshinone IIA [299], emodin, physcion [300], and aloe-emodin [301]. Idebenone exerts antioxidant effects that are mainly dependent on the benzoquinone ring, which has both reduced (hydroquinone) and oxidized forms [287]. The ketone bond can generate unstable semiquinone through a reduction reaction or further reduction to form dihydroubiquinone, which exhibits strong antioxidant activity. Hao Xu [294] examined the expression of SIRT3 in oxidative stress-injured HT22 cells before and after the use of ibuprofen and found that ibuprofen counteracted oxidative stress-injured neuronal apoptosis by affecting the CD38-SIRT3-P53 pathway. The optimal extraction process of naphthoquinones in water walnut leaves was determined by one-way and orthogonal tests, i.e., 50% *v*/*v* ethanol solution as extraction solvent, 1:50 (g/mL), extraction temperature of 60 °C, and extraction time of 5 h. The extraction of naphthoquinones reached 168.14 mg/g under these conditions. Hu Tian determined the optimal extraction process of naphthoquinone components in the leaves of *Platycarya strobilacea* Siebold & Zucc, through a single-factor and orthogonal experiment. That is, an ethanol solution with a volume fraction of 50% was used as the extraction solvent, with a solid-liquid ratio of 1:50 (g/mL), extraction temperature of 60 °C, and extraction time of 5 h. Under these conditions, the amount of naphthoquinone extract reached 168.14 mg/g. By measuring their reducing power, it was found that the DPPH radical scavenging ability of both the naphthoquinone extract of *Narcissus aquifolium* Pourr., and VC gradually increased with increasing sample mass concentration. However, the scavenging rate of DPPH radicals by both the naphthoquinone extract of *Narcissus aquifolium* Pourr., and VC gradually stabilized when the mass concentration of the naphthoquinone extract of *Narcissus aquifolium* Pourr., and VC was greater than 0.6 mg/mL. The results indicated that the naphthoquinone constituents of water walnut leaves have good antioxidant activity in vitro [302]. Table 8 introduces the antioxidant activity of quinone compounds.

### 3.4. Anti-Inflammatory Activity

Anthraquinones have significant anti-inflammatory effects, and their mechanism of action mainly involves the regulation of inflammatory factors and the inhibition of related signaling pathways. Through in vivo experiments in mice, Jie found that alcohol extracts of *Rubia cordifolia* L. exert anti-inflammatory effects by inhibiting the production of pro-inflammatory factors in serum and promoting the production of anti-inflammatory factors. *Rubia cordifolia* L. alcohol extract in the middle concentration group and high concentration group had similar therapeutic effects to that of dexamethasone on adjuvant arthritis in mice, resulting in a reduction in inflammatory cell infiltration in the articular cavity of the ankle joint in mice. The MDA and SOP levels in liver homogenates showed that the components in *Rubia cordifolia* L. inhibit inflammation partly through the elimination of free radicals and reactive oxygen molecules in vivo and partly through the metabolism of glutathione in the liver [303]. Liu Mingxin demonstrated that the naphthoquinone constituents of *Arnebia euchroma* (Royle) I. M. Johnst. were able to downregulate the expression of inflammatory mediators PGE2, NO, and inflammatory cytokines IL-1β and TNF-α, inhibit xylene-induced mouse auricular swelling, and exert certain anti-inflammatory effects in vitro using a macrophage inflammation model and in vivo in an animal model [304].

### 3.5. Antimicrobial Activity

Quinones have significant antimicrobial effects and inhibit a wide range of bacteria to varying degrees [305]. Their inhibitory mechanism mainly lies in their ability to inhibit the oxidation and dehydrogenation processes of bacterial sugars and metabolic intermediates, and they can bind to DNA, interfering with its template function, and thus inhibiting the synthesis of proteins and nucleic acids [306]. Zhenkang Lu treated *E. coli* with juglone at concentrations of 0.0625, 0.125, 0.25, 0.5, 1, 2 mg/mL, and 4 mg/mL, and the relative conductivity of *E. coli* cell membranes increased which means that juglone resulted in impaired integrity of *E. coli* membranes, and increased permeability of cell membranes. Fluorescence emission spectroscopy results showed that juglone interacts with membrane proteins, thereby changing the structure of the *E. coli* cell membrane. The results of crystal violet and bladed azurite staining experiments showed that juglone could weaken the respiration of *E. coli* by inhibiting the formation of *E. coli* biofilms and eventually inhibiting its activity. SDS—PAGE and *E. coli* genome synthesis analysis revealed that juglone inhibited the expression of proteins, DNA, and RNA in E. coli, thereby acting as an antibacterial agent [307].

### 3.6. Anti-Fibrotic Effect

Quinones have antifibrotic effects. One of these mechanisms involves the inhibition of fibrosis-related cytokine expression, interference with signaling pathways, and reduction of extracellular matrix synthesis. Anthraquinone can inhibit the over-activation of the MAPK pathway in hepatic stellate cells, thereby inhibiting the activation of hepatic stellate cells, reducing their transformation to myofibroblasts, reducing the synthesis of extracellular matrix, and reducing the degree of liver fibrosis. In vitro antifibrotic tests on rat HSC were performed, and it was concluded that 2,3,5-trihydroxy-4,9-dimethoxyphenanthrene, 2,3,5-trihydroxy-4-methoxyphenanthrene, and denbinobin phenanthrenequinone from Dendrobium officinale could all reduce the number of HSC cells. These three phenanthrenes exhibit antifibrotic activity by inducing the selective death of hematopoietic stem cells, providing a new avenue for the prevention and treatment of liver fibrosis [308].

### 3.7. Laxative Effect

Quinone compounds have purgative effects. The primary action sites of the combined anthraquinones of *Rhei Radix* or the free anthraquinones of *Rheum palmatum* L. *Radix* are the small intestine and stomach, followed by the colon. It can be seen that the anthraquinones of *Rheum palmatum* L. Radix et Rhizoma can act directly without the need for transformation in the large intestine [309]. Chen Yan-Yan [310] showed that after administration of Da Huang Gan Cao Tang to constipated mice, the time to peak and the area under the drug-time curve of the plasma anthraquinones emodin, aloe emodin, emodin-8-O-β-D-glucoside, conjugated dianthrones senecioside A, and glycyrrhetinic acid were higher than those of control mice. Compared to normal mice, rhubarb-glycyrrhiza glabra soup exhibited a stronger purifying effect in constipated mice, with an increase in fecal excretion and a shorter time to the first detachment.

### 3.8. Antidepressant Effects

Anthraquinones from medicinal plants, such as chrysin, also have antidepressant activity and are often used in antidepressant therapy. Chrysin has been found to improve depressive symptoms in rats, and high doses of chrysin can activate 5-hydroxytryptamine receptors (5-HT) in the hippocampus of depressed rats, stimulate neurotransmitter transmission, and increase the degree of excitability in rats. This anthraquinone analog reduced the degree of depression in rats [118].

## 4. Progress in Toxicity Studies

### 4.1. Digestive System Toxicity

#### 4.1.1. Hepatotoxicity

As exogenous substances, the main chemical components of quinones are oxidized and reduced under the action of the cytochrome P450-based monooxygenase system in the liver and are finally converted into polar compounds for excretion [311]. Hu Xichen conducted three consecutive months of gavage and histopathological examination of the rat liver. At the end of three months, histopathological sections of the liver showed scattered inflammatory cell infiltration, congestion of hepatic sinusoids, active proliferation of Kupffer cells, and phagocytosis of pigment particles under a light microscope. In the transmission electron microscopy of the high-dose group, chromatin was clumped together in the nuclei of some hepatocytes or collected in the subnuclear membranes, the mitochondria were mildly swollen, the structure of capillary bile ducts was not clear in individual specimens, and the number of Kupffer cells was increased. In some specimens, the structure of the capillary bile ducts was unclear, and the number of Kupffer cells was increased. After the recovery period, no obvious pathological changes were observed in the liver pathology section under the microscope in each administered group. The results demonstrated that long-term gavage of prepared *Polygonum multiflorum* can cause liver inflammatory injury in rats, and the liver can be normalized after stopping the drug [312]. Z.H. Mao investigated the potential cytotoxicity and DNA-breaking effects of rhein, chrysophanol, emodin methyl ether, and aloe emodin on HepaRG in normal human-derived hepatocytes by using high-concentration assay and alkaline comet electrophoresis. The four rhubarb anthraquinones were found to be toxic to hepatocytes to varying degrees. Among them, the effects of emodin methyl ether on elevated reactive oxygen species and mitochondrial damage were more pronounced, and the toxicity of aloe emodin was mainly manifested by the modulation of free Ca^2+^ levels in hepatocytes. Oxidative stress injury may be an important molecular mechanism responsible for potential hepatocytotoxicity and genotoxicity [313].

#### 4.1.2. Enterotoxicity

Quinones usually exist as glycosides and are not degraded by gastric acid. When anthraquinones is administered orally, it enters the stomach and small intestine through the esophagus, is absorbed into the bloodstream through the small intestinal mucosa, is converted to glucuronide conjugates by phase II enzymes in the liver and intestines [314], and is transported to various tissues and organs throughout the body through the heart to exert a variety of pharmacological effects [315]. Prolonged use of laxatives containing anthraquinones can cause colorectal melanosis (MC). MC is a non-inflammatory, benign, reversible pigmentation characterized by colorectal mucosal lesions [316], which has been found to be due to intestinal mucosal epithelial cellular turnover and deposition of lipofuscin by electron microscopy and histopathology. The presence of anthraquinone-containing laxatives in the colon significantly increases the risk of developing colorectal melanosis. SteerH W Ultrastructural and histochemical staining of colonic tissues from six normal colons and seven patients with melanotic polyps revealed that anthraquinone laxatives increased the number of macrophages in the lamina propria of the colonic mucosa. In addition, they enhance the lysosomal activity of macrophages, Schwann cells, and neuronal cells in the lamina propria of the colonic mucosa, as well as increase the number of lysosomes [317].

Cheng Ying used acridine orange staining and mitochondrial membrane potential staining to detect the effects of rhubarb sap metabolites, rhein, emodin, and aloe emodin on the acidic vesicular organelles and mitochondrial membrane potential in NCM460 and HT29 cells, respectively, and the effects of autophagy and apoptosis-related proteins on the expression levels were detected by western blot. The results showed that rhubarb sap metabolites, rhein, emodin, and aloe emodin, induced autophagy and apoptosis in NCM460 and HT29 cells, suggesting that rhubarb may exert a toxic effect on human colon cells by promoting autophagy and apoptosis [318].

### 4.2. Urinary Toxicity

Quinones can cause proteinuria, oliguria, anuria, hematuria, and other symptoms, and long-term or large amounts of exposure may lead to acute and chronic nephritis, renal failure, and even uremia and other serious kidney diseases, seriously affecting the normal function of the urinary system and overall health of the body. When emodin is ingested in excess, it interferes with the filtration function of the kidneys and the reabsorption of the renal tubules, resulting in the excretion of protein components that should have been reabsorbed back into the bloodstream through urine, leading to proteinuria. Lan Jie observed the effects of anthraquinone components in *Pleuropterus multiflorus* (Thunb.) Nakai on human renal cortical proximal tubule epithelial cell line HK-2 cells and detected the changes in mitochondrial membrane potential of HK-2 cells using JC-10. The mitochondrial membrane potential of the five anthraquinone monoconstituents declined with an increase in the treatment concentration and prolongation of the administration time, among which chrysophanin and aloe emodin had the fastest rate of decline, followed by rhodochrospiracol. The apoptosis of HK-2 cells after the administration of the five anthraquinone monomer components were detected by flow assay, and it was found that significant apoptosis was visible only after the administration of Rhein, Aloe emodin greater than or equal to 25 μmol/L for 48 h and and and Rhein 50 and 100 μmol/L for 48 h (*p* < 0.05). It was concluded that emodin, Aloe emodin, and Rhein can damage HK-2 cells with a potential risk of nephrotoxicity [319].

### 4.3. Reproductive Toxicity

Quinones may have adverse effects on the uterus and placenta during pregnancy. They cross the placental barrier and exert direct toxic effects on the fetus. Chang determined that emodin induced apoptosis, i.e., embryonic cytotoxicity, in mouse blastocysts by treating them with 25, 50, or 75 μmol/L emodin for 24 h at 37 °C and examining DNA fragmentation using the TUNEL assay. Membrane-associated protein V staining revealed a significantly higher number of membrane-associated protein V-positive/PI-negative (apoptotic) cells in the ICM and TE of emodin-treated blastocysts than in the control group. Emodin significantly inhibited cell proliferation and induced apoptosis in the ICM and TE of mouse blastocysts. Selective inhibition of RAR activity in emodin-treated blastocysts. Therefore, this substance may negatively affect embryonic development by decreasing RARβ expression, which in turn downregulates the RARβ-mediated developmental signaling pathways. Emodin triggers apoptosis in mouse blastocysts, leading to impaired embryonic development via the intrinsic cell death pathway [320].

Quinones can interfere with the normal physiological processes of testicular spermatogenic cells and damage DNA in sperm cells, causing gene mutations or chromosomal aberrations, thereby reducing the quality and quantity of spermatozoa. N-(1,3-Dimethylbutyl)-N′-phenyl-p-phenylenediamine (6PPD) is acutely toxic to organisms. Yao Kezhen exposed C57Bl/6 male mice to 6PPD-Q for 40 days at a dose of 4 mg/kg bw. After 40 days of exposure to C57Bl/6 male mice, exposure to 6PPD-Q not only resulted in decreased testosterone levels but also adversely affected semen quality and in vitro fertilization (IVF) results, thus indicating that 6PPD-Q exposure leads to impaired male fertility [321].

### 4.4. Carcinogenicity

The International Agency for Research on Cancer (IARC) of the World Health Organization (WHO) classifies carcinogens into five groups, of which quinones are classified as group 2B and group 3 carcinogens. The IARC classifies 1-amino-2,4-dibromoanthraquinone, anthraquinone, dantron (chrysazin; 1,8-dihydroxyanthraquinone), 1-hydroxyanthraquinone, 2-methyl-1-nitroanthraquinone (uncertain purity), and mitoxantrone as Group 2B carcinogens, i.e., possibly carcinogenic to humans, but evidence of carcinogenicity in humans is limited. Limited evidence of carcinogenicity in humans and insufficient evidence of carcinogenicity in experimental animals, or insufficient evidence of carcinogenicity in humans and sufficient evidence of carcinogenicity in experimental animals. 1-amino-2-methylanthraquinone, 2-aminoanthraquinone, and aziridyl benzoquinone are classified as Group 3 carcinogens, i.e., their carcinogenicity to humans is doubtful, and there are insufficient human or animal data. Table 9 introduces carcinogenic quinone compounds and their classifications.

## 5. Summary

Summarizing and analyzing the research literature at home and abroad, the current research on quinones focuses on their types, pharmacological activities, and toxicity, and abundant research results have been achieved in these fields. The application and potential risks of quinones in the field of medicine and health have been investigated from the perspectives of classification of chemical structure, verification of biological activity, and exploration of toxicity mechanism, which provides a rich theoretical basis and practical experience for the development of quinones in natural medicine and related products. However, it should be pointed out that this study also has certain limitations. For example, in toxicity research, the molecular mechanism of quinone toxicity remains unclear; in technical terms, the efficiency of extraction and separation techniques is limited, and structural identification is overly dependent on traditional methods. Based on the limitations of the current study, further systematic research can be carried out in the following aspects: firstly, a systematic toxicity evaluation of quinones in traditional Chinese medicine and risk assessment to evaluate the safety of these ingredients; secondly, with the help of a 3D organoid co-culture model, further in-depth investigation into the toxicity mechanism of quinones, clarifying the key links and molecular mechanisms of their toxicity, and synthesizing quinone compounds with high bioactivity and low toxicity. We will synthesize quinone derivatives with high biological activity and low toxicity to expand the application potential of quinone compounds. Finally, we will develop an efficient and accurate online identification technology for the rapid identification of quinone compounds in traditional Chinese medicine.

## Figures and Tables

**Table 6:** Names and molecular formulas of dianthrone compounds.

No.	Name	Resource	Formula	Type	Ref.
481	polygonumnolide C1	*Pleuropterus multiflorus* (Thunb.) Nakai	C_36_H_32_O_13_	type I	[238]
482	polygonumnolide C2	*Pleuropterus multiflorus* (Thunb.) Nakai	C_36_H_32_O_13_	type I	[238]
483	polygonumnolide C3	*Pleuropterus multiflorus* (Thunb.) Nakai	C_36_H_32_O_13_	type I	[238]
484	polygonumnolide C4	*Pleuropterus multiflorus* (Thunb.) Nakai	C_36_H_32_O_13_	type I	[238]
485	trans-emodin dianthrones	*Pleuropterus multiflorus* (Thunb.) Nakai	C_30_H_22_O_8_	type I	[238]
486	cis-emodin dianthrones	*Pleuropterus multiflorus* (Thunb.) Nakai	C_30_H_22_O_8_	type I	[238]
487	(+)-crinemodin-rhodoptilometrin dianthrone	*Himerometra magnipinna* AH Clark	C_35_H_32_O_8_	type I	[239]
488	7,7′-dichlorohypericin	*Heterodermia obscurata* (Nyl.) Trevis.	C_30_H_14_Cl_2_O_8_	type I	[240]
489	nephrolaevigatin A	*Nephroma laevigatum* Ach.	C_30_H_20_Cl_2_O_8_	type I	[241]
490	nephrolaevigatin B	*Nephroma laevigatum* Ach.	C_30_H_20_ClO_8_	type I	[241]
491	bioanthrone 1	*Vismia guineensis* (L.) Choisy	C_50_H_54_O_8_	type I	[242]
492	flavoobscurin B	*Heterodermia obscurata* (Nyl.) Trevis.	C_30_H_19_Cl_4_O_8_	type I	[241]
493	8,8′-dihydroxy-1,1′,3,3′-tetramethoxy-6,6′-dimethyl-10,10′-dianthrone	*Aspergillus wentii* Wehmer	C_34_H_30_O_8_	type I	[243]
494	hypericin	*Hypericum monogynum* L.	C_30_H_16_O_8_	type I	[244]
495	pseudohypericin	*Hypericum monogynum* L.	C_30_H_16_O_9_	type I	[244]
496	neobulgarone E	*Limonium tubiflorum* (Delile) Kuntze	C_32_H_24_Cl_2_O_8_	type I	[245]
497	polygonumnolide A1	*Pleuropterus multiflorus* (Thunb.) Nakai	C_37_H_34_O_13_	type II	[246]
498	polygonumnolide A2	*Pleuropterus multiflorus* (Thunb.) Nakai	C_37_H_34_O_13_	type II	[246]
499	polygonumnolide A3	*Pleuropterus multiflorus* (Thunb.) Nakai	C_37_H_34_O_13_	type II	[246]
500	polygonumnolide A4	*Pleuropterus multiflorus* (Thunb.) Nakai	C_37_H_34_O_13_	type II	[246]
501	polygonumnolide B1	*Pleuropterus multiflorus* (Thunb.) Nakai	C_43_H_44_O_18_	type II	[246]
502	polygonumnolide B2	*Pleuropterus multiflorus* (Thunb.) Nakai	C_43_H_44_O_18_	type II	[246]
503	polygonumnolide B3	*Pleuropterus multiflorus* (Thunb.) Nakai	C_43_H_44_O_18_	type II	[246]
504	polygonumnolide E	*Pleuropterus multiflorus* (Thunb.) Nakai	C_37_H_34_O_13_	type II	[247]
505	adamadianthrone	*Psorospermum febrifugum* Spach	C_45_H_46_O_8_	type II	[154]
506	bioanthrone 2	*Vismia guineensis* (L.) Choisy	C_30_H_20_O_11_	type II	[242]
507	glaberianthrone	*Psorospermum glaberrimum* Hochr.	C_45_H_46_O_8_	type II	[248]
508	prinoidin-emodin dianthrones	*Rhamnus napalensis* (Wall.) Lawson	C_40_H_37_O_14_	type II	[249]
509	(S)-2-hydroxybutyl-4,4′,5,5′,7-pentahydroxy-2′-methoxy-2,7′-dimethyl-10,10′-dioxo-9,9′,10,10′-tetrahydro-[9,9′-bianthracene]-3-carboxylate	*Aspergillus wentii* Wehmer	C_36_H_32_O_11_	type II	[249]
510	(S)-2-hydroxybutyl 4,4′,5,7-tetrahydroxy-5′,7′-dimethoxy-2,2′-dimethyl-10,10′-dioxo-9,9′,10,10′-tetrahydro-[9,9′-bianthracene]-3-carboxylate	*Aspergillus wentii* Wehmer	C_37_H_34_O_11_	type II	[249]
511	2,4′,5-trihydroxy-4,5′,7′-trimethoxy-2′,7-dimethyl-[9,9′-bianthracene]-10,10′(9H,9′H)-dione	*Aspergillus wentii* Wehmer	C_33_H_28_O_8_	type II	[249]
512	dianthrone A1	*Psorospermum febrifugum* Spach	C_50_H_54_O_8_	type III	[154]
513	bioanthrone 3	*Vismia guineensis*	C_30_H_20_O_12_	type III	[242]
514	dianthrone A2a	*Psorospermum glaberrimum* Hochr.	C_45_H_46_O_8_	type III	[242]
515	dianthrone A2b	*Psorospermum glaberrimum* Hochr.	C_40_H_38_O_8_	type III	[248]
516	prinoidin dianthrones rhamnepalins	*Rhamnus napalensis* (Wall.) M.A.Lawson	C_50_H_51_O_20_	type III	[249]
517	8,8′-dihydroxy-1,1′,3,3′-tetramethoxy-6,6′-dimethyl-10,10′-dianthrone	*Aspergillus wentii* Wehmer	C_34_H_30_O_8_	type III	[243]
518	physcion-10,10′-bianthrone	*Cassia didymobotrya* Fresen.	C_32_H_28_O_8_	type III	[250]
519	dianthrone J	*Cratoxylum formosum* subsp. *pruniflorum* (Kurz) Gogelein	C_42_H_42_O_8_	type III	[251]
520	(−)-trans-2,2′-Digeranyloxy-7,7′-dimethyl-4,4′,5,5′-tetrahydroxy-9,9′-dianthrone	*Ochna pulchra* Hook.	C_50_H_54_O_8_	type III	[252]
521	trans aloe-emodin dianthrone diglucoside	*Cassia angustifolia* Vahl	C_42_H_42_O_18_	type IV	[253]
522	sennoside B	*Senna alexandrina* Milll.	C_42_H_38_O_20_	type V	[254]
523	(−)-ochnadianthrone	*Ochna pulchra* Hook.	C_50_H_54_O_8_	type V	[255]
524	sennidin C	*Rheum palmatum* L.	C_30_H_20_O_9_	type VI	[255]
525	sennoside A	*Senna alexandrina* Milll.	C_42_H_40_O_19_	type VI	[254]
526	sennoside D	*Senna alexandrina* Milll.	C_48_H_44_O_25_	type VI	[256]
527	sennoside E	*Senna alexandrina* Milll.	C_48_H_44_O_25_	type VI	[254]
528	sennoside F	*Senna alexandrina* Milll.	C_48_H_44_O_25_	type VI	[254]
529	chrysophanol dianthrone	*Heterodermia obscurata* (Nyl.) Trevis.	C_30_H_21_O_6_	type VII	[240]
530	chrysophanol-l0,l0′-dianthrone	*Cassia didymobotrya* Fresen.	C_30_H_22_O_6_	type VII	[250]
531	chrysophanol-isophyscion dianthrone	*Senna longiracemosa* (Vatke) Lock	C_31_H_25_O_7_	type VII	[257]
532	isophyscion dianthrone	*Senna longiracemosa* (Vatke) Lock	C_32_H_28_O_8_	type VII	[257]
533	martianine 1	*Senna martiana* (Benth.) H. S. Irwin & Barneby	C_43_H_44_O1_6_	type VII	[258]
534	palmidin B	*Rheum palmatum* L.	C_30_H_22_O_7_	type VII	[258]
535	palmidin C	*Rheum palmatum* L.	C_30_H_22_O_7_	type VIII	[259]
536	neobulgarone G	*Limonium tubiflorum* (Delile) Kuntze	C_32_H_24_Cl_2_O_9_	other	[245]
537	chrysophanol-physcion-l0,l0′-dianthrone	*Cassia didymobotrya* Fresen.	C_31_H_25_O_7_	other	[250]
538	1,8,1′,8′-tetrahydroxy-10,10′-dianthrone	*Hypericum* Tourn. ex L.	C_28_H_18_O_6_	other	[260]
539	palmidin A	*Rheum palmatum* L.	C_30_H_22_O_8_	other	[259]
540	rendin A	*Rheum palmatum* L.	C_30_H_20_O_9_	other	[255]
541	rendin B	*Rheum palmatum* L.	C_30_H_20_O_8_	other	[255]
542	rendin C	*Rheum palmatum* L.	C_31_H_22_O_9_	other	[255]

**Table 7:** Anti-proliferative effects of quinone compounds on cells.

No.	Name	Cell Line	IC50
15	embelin	PC-3	3.7 μmol/L
		LNCaP	5.7 μmol/L
		HeLa	5–7 μmol/L
64	juglone	BxPC-3	21.05 μmol/L
68	plumbagin	HepG2	(27.08 ± 0.40) μmol/L
		HL-60	0.8 μmol/L
197	dioscoreanone	MCF-7	20 μmol/L
198	denbinobin	K562	1.84 μmol/L
		GSK5182	1.6 μmol/L
219	tanshinone IIA	A549	42.45 μmol
		BGC-823	61.46 μmol/L
		Hep-2	9.6 μmol/L
226	chrysophanol	HepG2	30 μmol/L
		MCF-7	25 μmol/L
		A549	18 μmol/L
227	emodin	HL-60/ADR	5.79 μmol/L
		SMMC-7721	21.6 μmol/L
		HL-60	20 μmol/L
		L02	135 μmol/L
521	aloe-emodin	HeLa	58.3 μmol/L
		HepG2	10 μmol/L
		HCT116	8.7 μmol/L

**Table 8:** Antioxidant activity of quinone compounds.

No.	Name	DPPH	ABTS
68	plumbagin	IC50 = 50 μmol/L	
64	juglone	IC50 = 0.498 mg/mL	IC50 = 0.189 mg/mL
142	alkannin	IC50 = 40 μg/mL	
217	tanshinone I	IC50 = 0.07 μmol/L	
227	emodin	EC50 = 147.87 mg/L IC50 = 112.32 mg/mL	
385	physcion	IC50 = 56.05 mg/mL	
521	aloe-emodin	EC50 = 6.03 mg/L	

**Table 9:** Carcinogenic quinone compounds and their classification.

No.	Name	Classification
225	dantron(chrysazin;1,8-dihydroxyanthraquinone)	2B
328	1-hydroxyanthraquinone	2B
336	1-amino-2,4-dibromoanthraquinone	2B
337	2-methyl-1-nitroanthraquinone	2B
397	mitoxantrone	2B
59	tris(aziridinyl)-para-benzoquinone (triaziquone)	3
60	aziridyl benzoquinone	3
371	1-amino-2-methylanthraquinone	3
386	2-aminoanthraquinone	3

## Data Availability

The data presented in this study are available upon request from the corresponding author.
